# Development of Electrode-Supported Proton Conducting Solid Oxide Cells and their Evaluation as Electrochemical Hydrogen Pumps

**DOI:** 10.1021/acsami.2c11779

**Published:** 2022-08-18

**Authors:** Usman Mushtaq, Stefan Welzel, Rakesh K. Sharma, M.C.M. van de Sanden, Mihalis N. Tsampas

**Affiliations:** † Dutch Institute For Fundamental Energy Research (DIFFER), Eindhoven 5612AJ, The Netherlands; ‡ Department of Chemical Engineering and Chemistry, Eindhoven University of Technology, Eindhoven 5600 MB, The Netherlands; § Department of Applied Physics, Eindhoven University of Technology, Eindhoven 5600 MB, The Netherlands

**Keywords:** protonic ceramic solid oxide cell (P-SOC), Ni-BCZY electrode-supported cell, vacuum-assisted coating, pinhole-free BCZY electrolyte, electrochemical hydrogen pump

## Abstract

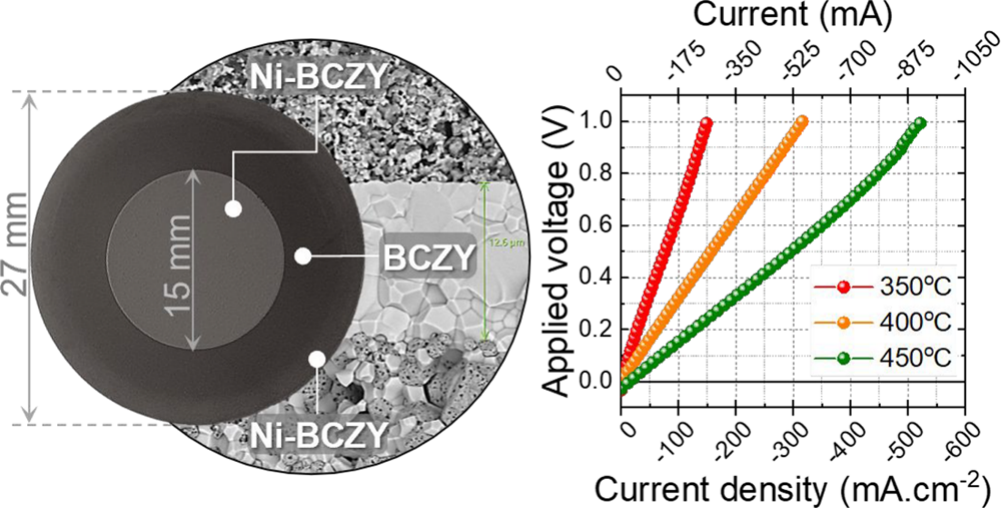

Protonic ceramic solid oxide cells (P-SOCs) have gained widespread attention due to their potential for operation in the temperature range of 300–500 °C, which is not only beneficial in terms of material stability but also offers unique possibilities from a thermodynamic point of view to realize a series of reactions. For instance, they are ideal for the production of synthetic fuels by hydrogenation of carbon dioxide and nitrogen, upgradation of hydrocarbons, or dehydrogenation reactions. However, the development of P-SOC is quite challenging because it requires a multifront optimization in terms of material synthesis and fabrication procedures. Herein, we report in detail a method to overcome various fabrication challenges for the development of efficient and robust electrode-supported P-SOCs (Ni-BCZY/BCZY/Ni-BCZY) based on a BaCe_0.2_Zr_0.7_Y_0.1_O_3−δ_ (BCZY271) electrolyte. We examined the effect of pore formers on the porosity of the Ni-BCZY support electrode, various electrolyte deposition techniques (spray, spin, and vacuum-assisted), and thermal treatments for developing robust and flat half-cells. Half-cells containing a thin (10–12 μm) pinhole-free electrolyte layer were completed by a screen-printed Ni-BCZY electrode and evaluated as an electrochemical hydrogen pump to access the functionality. The P-SOCs are found to show a current density ranging from 150 to 525 mA cm^–2^ at 1 V over an operating temperature range of 350–450 °C. The faradaic efficiency of the P-SOCs as well as their stability were also evaluated.

## 1 Introduction

In recent years, an extensive amount of CO_2_, one of the major components of greenhouse gases, has been released into the atmosphere due to anthropogenic activities. This has led to global climate change and possible ocean acidification.^1^ In this context, a change in the primary energy source from fossil fuels to so-called renewable energies such as, for instance, on-site hydrogen production from renewables offers great potential for fulfilling the growing energy demand and addressing environmental pollution problems.^2^ In fact, hydrogen can be used as a clean energy carrier to power fuel cell vehicles,^3,4^ produce electricity or heat,^5^ and store and transport surplus renewable energy. While it is a key feedstock in a number of existing chemical and refinery industrial processes, such as ammonia synthesis,^6^ oil refining and methanol production,^7^ the vast majority of industrial hydrogen is still produced from steam methane reforming (SMR)^8^ and coal gasification^9^ – gray hydrogen production inevitably leads to byproducts of CO, CO_2_, CH_4_, and H_2_O. Hydrogen produced from water electrolysis—green hydrogen—contributes less than 0.1% to global hydrogen production and is also twice as expensive as SMR.^8,10^

This calls for the separation of pure hydrogen from the product gases of SMR, which can be realized via a number of technologies, such as pressure swing adsorption^11−13^ or through the use of a galvanic hydrogen pump employing a perovskite based protonic ceramic membrane.^14−16^ For the former techniques, hydrogen permeation occurs under a high pressure gradient across a selective metal-based membrane (usually composed of palladium or its alloys with a thickness of 10–15 μm) or molecular sieves.^12^ Although an established hydrogen purification technology operates well at large scales with a high pressure gradient (5–20 bar) across the membrane, it is capital and energy intensive. However, it also remains challenging to selectively purify or sieve hydrogen out of carbonaceous streams at a higher rate, especially at low concentrations and atmospheric feed pressures, while also avoiding hydrogen embrittlement,^17^ competitive adsorption between hydrogen and methane or nitrogen or retaining the thermochemical integrity. The galvanic hydrogen pump^18^ is another application of the protonic solid oxide cells (P-SOCs), also referred to as protonic ceramic cells (PCCs). They are based on the direct electrochemical dissociation of hydrogen (eq 1) from the feed at the positive electrode (positrode^19^), transport of protons through a perovskite-based protonic ceramic membrane while electrons pass through an external circuit, followed by recombination (eq 2) of protons to evolve as pure hydrogen on the negative electrode (negatrode^19^) upon application of a DC current.
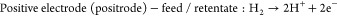
1
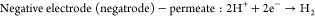
2Despite the simplicity of its nature, the development of such a P-SOC has been slow or limited to lab-scale prototypes (effective surface area <1 cm^2^)^15^ due to various challenges, notably including large area defect-free membrane processing, the requirement for high sintering temperatures, chemo-thermomechanical compatibility with the components, and the necessity for gastight sealing and interconnection components to upscale to a stack level.^15,20−22^ Importantly, the key parameters that govern the performance of a P-SOC pump are the conductivity, thickness, and gas-tightness of the membrane, while other parameters are kept constant (e.g., temperature, partial pressure of hydrogen, etc.).

For instance, reducing the membrane thickness decreases the ohmic losses and improves performance but also generates lower tensile stresses at the membrane–electrode interface during sintering processes^23^ when compared to a thicker membrane. However, a thin (5–20 μm) ceramic membrane is often very brittle in nature and generally requires mechanical support to function. A relatively thicker (250–800 μm) electrode with mixed protonic-electronic conductivity and high mechanical strength often acts as the support in this case. The requirements for a membrane are well described in refs (24 and 25). If we consider the highly conductive membrane material (Y-doped BaCeO_3_), it is structurally resilient but chemically unstable in humid or CO_2_-rich environments, limiting its use as a pump. To address this issue, there has been progress in improving the stability of this material by partially substituting Ce^4+^ with Zr^4+^. By doing so, multiple compositions of doped BaCe_1–*x*–*y*_Zr_*x*_Y_*y*_O_3−δ_ (BCZY)^26−30^ have been proposed to be suitably conductive and stable under the desired environments. Unfortunately, membranes with Zr^4+^-rich compositions exhibit poor mechanical strength and require higher sintering temperatures for grain growth to improve bulk conductivity.^31,32^ Techniques such as pulsed laser deposition (PLD) and atomic layer deposition (ALD) have been employed to overcome this issue and achieve highly conductive and extremely thin and dense BZY membranes that can be deposited onto Si^33^ or MgO^34^ planar substrates. However, these membranes are either free-standing, limited to a smaller membrane area, or suffer from pinhole formation when deposited onto a porous electrode.^35^ To fabricate a gastight membrane, PLD over a graded functional layer^36^ has been successfully carried out only after prior successive coating and refining steps. These techniques, although promising, have not yet been upgraded for larger area working devices or stacks.

Alternately, densifying the membrane for gas tightness during the formation of the perovskite phase can also be achieved via a solid-state reactive sintering (SSRS)^37^ approach where the stoichiometry and homogeneity of a polycrystalline nature^38^ is ensured in a single heat treatment step. In this approach, the precursors for the desired stoichiometric ratio are deposited over a support electrode by either spray coating,^39^ tape casting,^40^ or screen printing.^41^ For devices based on a planar architecture, the SSR method requires an intermediate precalcination step to compensate for volume shrinkage due to densification and prevent warping^42^ of the layers as opposed to that for a tubular type of architecture, where the effect of densification and the resulting strain is radially balanced out. This approach, although it reduces the number of processing steps, can leave behind some unreacted precursor, giving rise to a mixed-phase membrane.^43^ Moreover, by giving sufficient time for the reaction of the precursors, the loss of BaO from the membrane to the transient phase has been reported, which is otherwise precompensated at the beginning. However, other inhomogeneities within the membrane can also be generated by the formation of intermediate phases, e.g., BaY_2_NiO_5_.^40^ In addition, the associated elevated sintering temperatures and longer soaking times turn out to be detrimental. To avoid material decomposition, researchers have used sintering aids such as NiO, ZnO, Co_2_O_3_, and CuO^38,44−46^ to promote densification and grain growth at lower temperatures. Although these methods have been successful in decreasing the temperature, they also result in the formation of other phases, e.g., Y_2_O_3_, decrease the overall bulk proton conductivity, and lead to accumulation of sintering elements at the grain boundaries or the possible introduction of electronic conductivity.^47−49^

Upon successful fabrication of the half-cell, i.e., electrode-supported membrane, device fabrication is completed by attaching an electrode over the membrane by screen printing^42^ or brush^50,51^ or dip coating.^52^ Physical adhesion is achieved with a lower temperature thermal treatment than that used for membrane densification. Once completed, it requires effective integration into a test apparatus for performance evaluation. For this purpose, a sealant (glass, ceramic, or mix) with a matching thermal expansion coefficient (TEC)^53^ and chemical compatibility is chosen to provide hermetic sealing with other components (cell–fixture or interconnects) to isolate the gas environment while retaining the integrity of the cell during heating or cooling. This unit cell assembly is further integrated into a stack using repetitive components (unit P-SOCs, interconnects, sealants, etc.) based on the desired design requirements. In short, the overall fabrication of P-SOCs is a multifront challenge from materials and fabrication perspectives, and thus requires optimization of the parameters for a given material and architecture.

In this contribution, we present an asymmetrical (in terms of structure and geometry) P-SOC consisting of a dense protonic ceramic electrolyte (BaCe_0.2_Zr_0.7_Y_0.1_O_3-δ_ (BCZY271), ∼10–12 μm) that is deposited over a cermet electrode (40 wt % BCZY271–60 wt % NiO, ∼550 μm) and screen-printed with (40 wt % BCZY271–60 wt % NiO, ∼15 μm, 1.75 cm^2^) a similar electrode. We discuss in detail the fabrication steps and the parameters that affect the optimization of the support electrode and coating technique-specific thin and dense membrane without the need for sintering aids and higher temperature treatments. Here, the membrane was prepared by a vacuum-assisted dip coating technique, resulting in a densely packed material embedded onto a porous nickel-BCZY271 support electrode that was sintered between 1300 and 1500 °C at various sintering times. We assessed the fabricated layers at critical stages by using SEM, XRD, and EDX. Finally, the asymmetric P-SOC was evaluated electrochemically as a hydrogen pump to validate its functionality in the intermediate temperature range, i.e., 350–450 °C.

The proposed fabrication pathway leads to efficient P-SOCs, which suggests that appropriate modifications can be the stepping stone for implementing a series of essential electrochemical reactions for energy storage or conversion. In particular, with suitable screen-printed electrodes, the as-prepared half-cells can be used for a variety of applications owing to their chemical stability in steam and carbon dioxide environments. P-SOCs operating in the intermediate temperature range are instrumental for hydrogen production from ammonia or methane,^54,55^ steam electrolysis,^42,56^ cogeneration of energy and chemicals,^57,58^ ammonia synthesis,^59,60^ carbon dioxide valorisation,^51,61^ electrochemical promotion of catalysts,^62,63^ or hydrocarbon valorisation.^64^

## 2 Experimental Section

### 2.1 Materials

See Table 1.

**Table 1:** List of Materials and Suppliers

material	supplier	material	supplier
BCZY271 powder	cerpotech.com	dextrin type II	sigmaaldrich.com
NiO powder	fuelcellmaterials.com	dibutyl phthalate	sigmaaldrich.com
ink vehicle	fuelcellmaterials.com	Triton x-100	sigmaaldrich.com
gold paste	fuelcellmaterials.com	SN-dispersant 9228	sannopco.com
Iotect starch	nl.vwr.com	polyvinyl butyral	sekisui-sc.com
Prolabo starch	nl.vwr.com	gold mesh and wires	fiaxell.com
dextrin type I	sigmaaldrich.com	glass sealant	schott.com

### 2.2 Protonic Solid Oxide Cell (P-SOC) Preparation

The complete asymmetrical P-SOC was fabricated in a stepwise approach as described in the following sections. The device asymmetry arises from the different areas and thicknesses of the support and printed electrode.

#### 2.2.1 Support Electrode (Positrode) Fabrication

**Figure 1:**
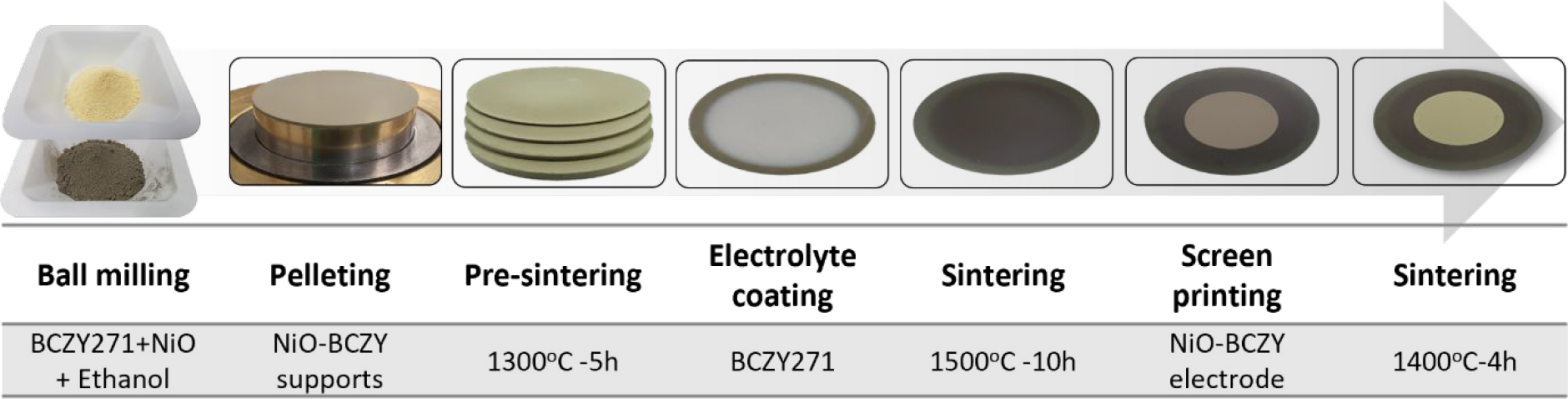
Fabrication process for an asymmetric protonic solid oxide cell (P-SOC) from precursor powders.

The cell is built upon the support electrode, referred to interchangeably as a positrode, which comprises BCZY271 and NiO powders, as illustrated in Figure. 1. BCZY271 and NiO powders were mixed in a 60:40 wt % ratio and ball-milled with zirconia balls (1, 3, and 5 mm) for 24 h at 400 rpm in ethanol. The mixture was crushed and sieved through 170 mesh (88 μm) to remove impurities after drying at 80 °C. A pore former (Prolabo–potato starch, dextrin from corn starch types I and II, and Iotect starch) of approximately 10 wt.% was separately added to the powder mixture. Two grams of the powder was weighed and transferred to the pelleting dye with a diameter of 35 mm and pressed at 150 MPa for 1 min using a uniaxial press (Atlas, Specac). Four pellets were stacked together and about 0.42 g cm^–2^ of dead weight was used to restrain them from warping. The pellets were immediately presintered at 1300 °C for 5 h, with an intermediate step to burnout the pore formers. The presintered supports were ground and polished with 5 and 10 μm silicon carbide (SiC) sandpaper to even the surface roughness, after which they were sonicated in ethanol for 10 min to remove the loose particles produced during the grinding step.

#### 2.2.2 Electrolyte Preparation and Coating

The electrolyte suspension was prepared in two steps: in the first step, polyvinyl butyral as a binder was added to the isopropanol and mixed with a magnetic stirrer until a clear liquid was obtained. Dibutyl phthalate, Triton X-100, and SN dispersant were added to the liquid as a plasticizer, surfactant, and dispersant. The solution was mixed until no residuals were visible. BCZY271 powder was added into a separate beaker and sonicated for 30 min at a power of 15 W using a Bendelin sonoplus HD4200. Both liquids were mixed together in a borosilicate bottle and pulsed sonicated for another 30 min while sitting on a magnetic stirrer at 100 rpm. The solid content of the BCZY271 powder was kept at 6 wt % in the suspension liquid.

The electrolyte was coated onto the support electrode by vacuum-assisted dip coating. The coating apparatus consisted of a vacuum pump, a digital pressure measurement gauge, a pressure regulator valve, and an aluminum jig. The jig was made to hold the electrode support over an O-ring via a regulated vacuum of 0.4–0.5 bar (300–380 mmHg). For coating, the electrode was immersed into the suspension for 30–60 s and then taken out to dry under atmospheric conditions. The process was repeated six to eight times to obtain the desired thickness for the coating layer. Upon drying in ambient air, two electrolyte coated support electrodes were sintered together by keeping the coated sides facing each other. A dead weight of 0.28 g cm^–2^ was used to retain the flatness throughout the sintering stage. They were sintered at 1300, 1450, and 1500 °C with an intermediate heating rate of 1 °C/min between 350 and 500 °C to burn out the organic components. In this way, the obtained thickness of a fully sintered electrolyte and the support was ∼12 and ∼550 μm with a diameter of 27 mm on average (Figure S1).

#### 2.2.3 Thin Electrode (Negatrode) Preparation and Coating

A powder mixture of NiO-BCZY271 in a 60:40 wt % ratio was added to a 40 wt % ink vehicle and ground to obtain a homogeneous paste using a mortar and pestle. The paste was screen printed (mesh 325–44 μm) onto the BCZY271 electrolyte twice with blow-drying after each printing step, and finally sintered at 1400 °C for 4 h. The active electrode area, average mass and thickness of the sintered electrode were approximately 1.75 cm^2^, 11 mg (6.3 mg cm^–2^) and 15 μm, respectively (Figure 1).

### 2.3 Physico-chemical Characterization

Scanning electron microscopy (Phenom-Pharos SEM) was employed to analyze the microstructural characteristics of the cell and to evaluate the integrity of the sintered layers. A detailed analysis of the morphology, porosity, grain, pore size and distribution was performed using *ImageJ* software. X-ray diffraction was performed to determine the phase purity and average grin size of the electrolyte material at various sintering temperatures and time durations. The XRD patterns (Cu Kα) were obtained by using a Bruker D8 eco in the Bragg–Brentano geometry. Nickel foil was used to filter out the Kβ part of the source radiation. A fixed membrane area of 1 cm × 1 cm was illuminated during the measurements. From the observed peak positions, the crystal texture was extracted. The width of the diffraction peaks contains information for the average grain size in that direction. To assess the crystal size (*d*), we used the Scherrer equation:
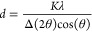
3where *K* (*K* = 1 in this study) is the dimensionless shape factor, λ is the X-ray wavelength for Cu Kα_1_ (λ=0.1504 nm), Δ(2θ) is the fixed width half maxima (fwhm) in units of 2θ and θ is the Bragg angle of the peak under consideration. Error data bars derived for Scherrer’s equation were calculated by using the fw ± error and were 0.5 times the measurement step size. The lattice parameter was determined using DIFFRAC.EVA software with the space group *Pm*3*m* of cubic symmetry. The Kα_2_ peaks were also removed using the same software.

### 2.4 P-SOC Evaluation

**Figure 2:**
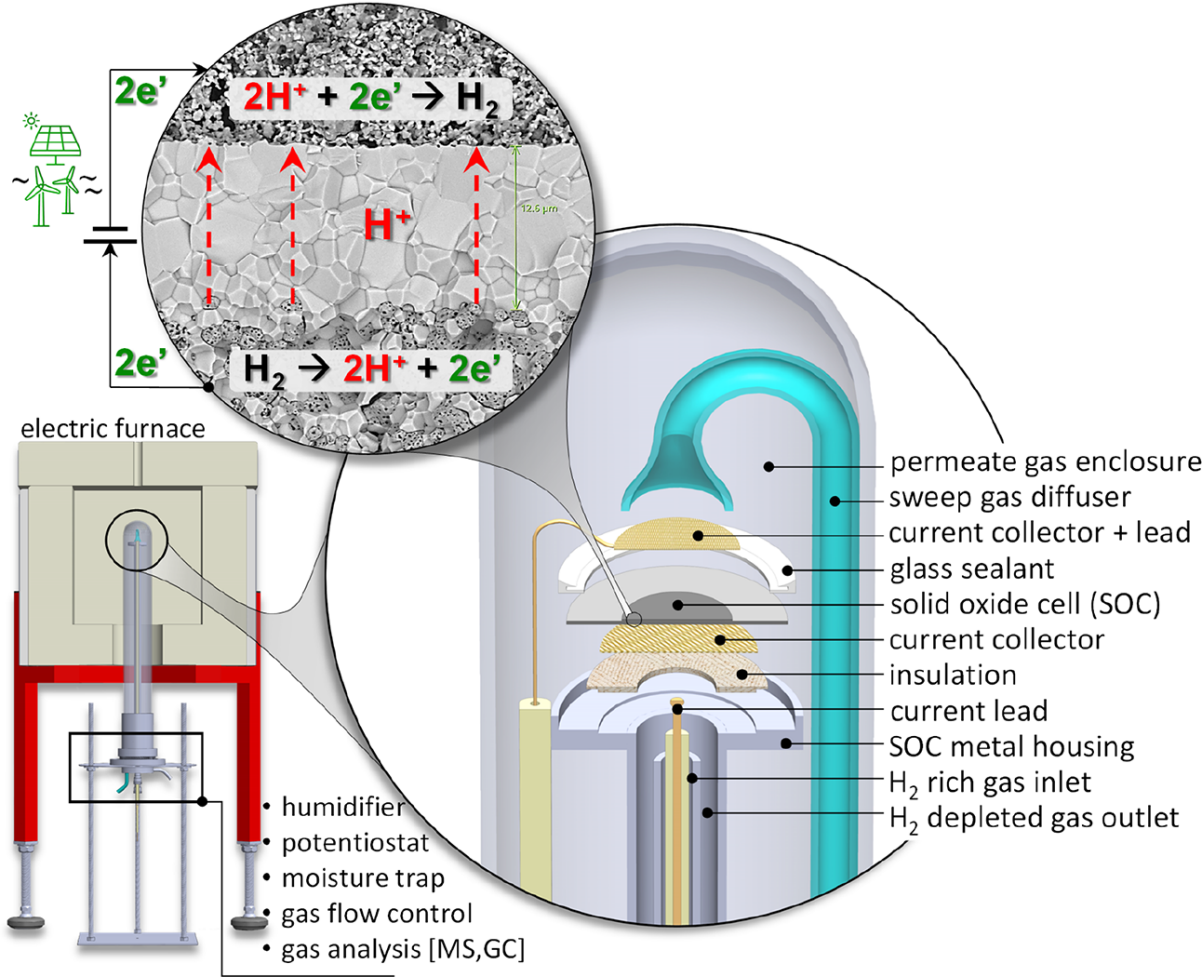
Proton conducting solid oxide cell test and evaluation setup.

The P-SOC was evaluated electrochemically after installing it in a test apparatus designed specifically to determine the properties of the cell. The fabricated P-SOC was installed such that the gas environments of either side of the electrolyte/membrane were completely isolated from each other and offer a real-time permeate gas analysis. For this purpose, the P-SOC was sealed to a metal housing with the help of a glass sealant, as shown in Figure S2. The housing was connected to the diagnostics underneath. Before sealing, gold mesh was attached to the positrode using a conductive gold paste as a current collector. A porous aluminosilicate layer was then placed between the positrode and metal housing for electrical insulation. To create a hermetic seal, a layer of the glass paste was applied over the electrolyte. The permeate side current collection was attached after curing the glass paste at 700 °C for 4 h under flowing air (∼100 sccm). A gold mesh was placed over the negatrode with the help of a sweep gas diffuser; the other side of the diffuser was harnessed at the bottom of the test apparatus and further connected to the gas flow control and conditioning apparatus (Figure 2). Electrical contact with the positrode was established (manually) after the P-SOC was heated to the operation temperature. An electric furnace (Bentrup TC 505) was used to heat and maintain the temperature of the cell, which was determined to range between 350 and 450 °C. A k-type thermocouple was placed near the cell to monitor the exact temperature.

The electrochemical characterization of the P-SOC, including data acquisition and control, was performed via a Vertex.5A. EIS (Ivium Technologies) potentiostat. The inlet gas flows (air, hydrogen, helium) on either side of the P-SOC were independently controlled by the designated thermal mass flow controllers (Brooks – GF 40 series). Hydrogen-rich feed gas was humidified by passing it through a water bubbler kept at 25 °C, supplying a flow rate of 5–15 cm^3^ min^–1^ to the positrode. A constant flow of humidified helium as a permeate sweep gas was maintained at the negatrode throughout the efficiency measurement experiment. A water trap (water eliminator) was installed at the permeate side to condense and trap the moisture. The evolved hydrogen concentration was monitored using a quadrupole mass spectrometer (HAL 201RC) and a gas chromatograph (Interscience, global analyzer solution, Compact GC 4.0) equipped with thermal conductivity and flame ionization detectors (TCD and FID).

Electrochemical impedance spectroscopy (EIS) was performed over the frequency range of 1 MHz to 1 mHz for a two-probe measurement setup with a perturbation amplitude of 10–15 mV at open circuit voltage (OCV) under a symmetric gas environment. The data were validated by the Kramers–Kronig (KK)^65^ compliance and the residuals in the real and imaginary parts deviating from the KK were calculated as follows:
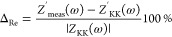
4
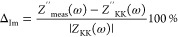
5The faradaic efficiency (FE) of the pump was determined by applying a constant current across the P-SOC for 30 min while measuring the concentration of hydrogen and the exhaust gas flow rate (in sccm) in the permeate side. The current steps were increased in increments to a maximum until a voltage of 1 V was reached. The evolved hydrogen flow rate (*V̇*_H_2_,evolved_) was divided by the amount of theoretically equivalent hydrogen (*V̇*_H_2_,theoretical_) to the current applied across the P-SOC by using the following expressions:
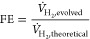
6
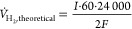
7where *I* is the applied current in ampere (C/s) and *F* is Faraday’s constant (96485 C mol^–1^). The rate unit from mol s^–1^ was converted to cm^3^ min^–1^ by multiplying 60 s min^–1^ and 24 000 cm^3^ mol^–1^, which corresponds to the reactor outlet conditions, i.e., at room temperature and ambient pressure. Any steady leaks were subtracted as background during the FE calculations. Gas diagnostic devices were calibrated using standard known gas compositions prior to usage. The cell was reduced for 24 h at 450 °C prior to operation; the reduction conditions and behavior are shown in Figure S3.

## 3 Results and Discussion

### 3.1 Effect of Pore Former on the Support Electrode

As per the literature,^66^ percolation of each phase is important, and as such, a 60:40 ratio of NiO:BCZY271 by weight was used to fabricate the support. For an ideal electrode, tortuosity (solid phase, pores), porosity, surface area, and triple phase boundaries (TPBs) are the key parameters that contribute to the electrical performance of an electrochemical cell. In fact, the porosity depends on the pore-former (content, types) and the quantity of the solid phases, e.g., NiO-BCZY, in this case. Therefore, we first investigated the effect of the pore former on the microstructure of the support (NiO-BCZY) sintered at 1300 and 1500 °C for 5 and 10 h. Along with these pore formers, we explored the required suitable compaction stress based on the pore-former content in the support. We found that ∼150 MPa is suitable for processing with 10 wt.% pore former content. A higher content, e.g., 20 wt %, will increase the porosity but require a higher compression of 375 MPa to maintain the mechanical strength for the 60:40 support composition.^41^

**Figure 3:**
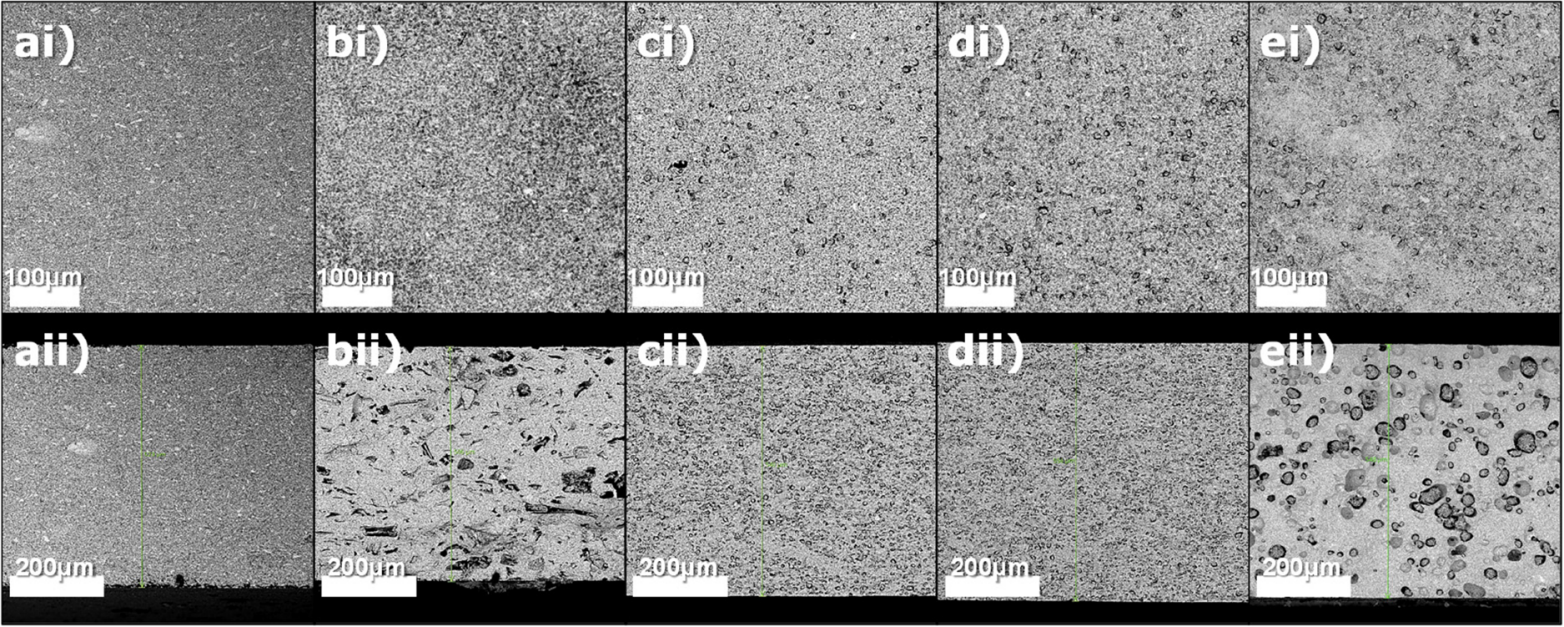
SEM images of a NiO-BCZY271 support electrode presintered at 1300 °C for 5 h with different types of pore formers: (ai) surface of the electrode without any pore former, (bi) with Iotect as a pore former, (ci) with dextrin I as a pore former, (di) with dextrin II as a pore former, (ei) with Prolabo as a pore former, and (aii–eii) the fractured cross-section images of the same samples.

As shown in Figure 3 and Table 2, different pore formers lead to pores of varying sizes and distributions. The support prepared without the addition of pore former shows negligible porosity when presintered at 1300 °C. The estimated microporosity is approximately 0.6%, which is also a clear indication of the absence of pores (Table 2 and Figures 3ai-ii). This will limit the electrochemical performance due to the mass transport limitation; moreover, in the absence of connected pores, the coating suspension is unable to seep through the substrate. Hence BCZY material deposition does not occur if the substrate is dense. Therefore, different pore formers were used to introduce the porosity. For example, Iotect and Prolabo (Figures 3bi and 3ei) result in a porous surface. However, the pore structures are irregular and gigantic, as observed in their cross-section images (Figures 3bii and 3eii). Both types generate macro porosities of 24.3 and 29%, with a microporosity ranging between 1.3 and 5.4% for the supports. This irregular feature is an undesired property in terms of mechanical strength and may generate point defects. Importantly, since the electrolyte suspension contains submicron particles, the chances of deposition only at the macropore are high. Thus, this type of porosity will not favor the method employed for electrolyte deposition. The supports with the pore formers dextrin I and II are of similar types, which is also depicted by their estimated porosities in Table 2. We chose dextrin II because it generates well-distributed macro- and micropores across the supports. The generated porosity decreases from 11.7 to 3.8% for samples sintered from 1300 to 1500 °C due to further densification. However, it is important to mention the increase in the porosity as the support is reduced (Figure S4). The macroporosity increases from 3.8 to 6%, but micropores are generated due to the reduction of solid nickel agglomerates into sponge-like particles, which give rise to an overall porosity and hence better gas diffusion through the support electrode.

**Table 2:** Estimated Porosity Features of the Support Electrode with Varying Pore Former Types, Sintering Temperature, and Treatment

pore former (PF) type	without PF	Iotect	dextrin I	Prolabo	dextrin II	dextrin II	dextrin II
sintering temp. (°C)	1300	1300	1300	1300	1300	1500	1500
treatment	unreduced (NiO-BCZY)	unreduced (NiO-BCZY)	unreduced (NiO-BCZY)	unreduced (NiO-BCZY)	unreduced (NiO-BCZY)	unreduced (NiO-BCZY)	reduced (Ni-BCZY)
porosity_macro_ (%)		24.3	10.8	29.0	11.7	3.80	6.00
porosity_micro_ (%)	0.60	1.30	3.70	5.40	3.80		22.9

### 3.2 Electrolyte Coating Characterization

#### 3.2.1 Effect of Temperature

**Figure 4:**
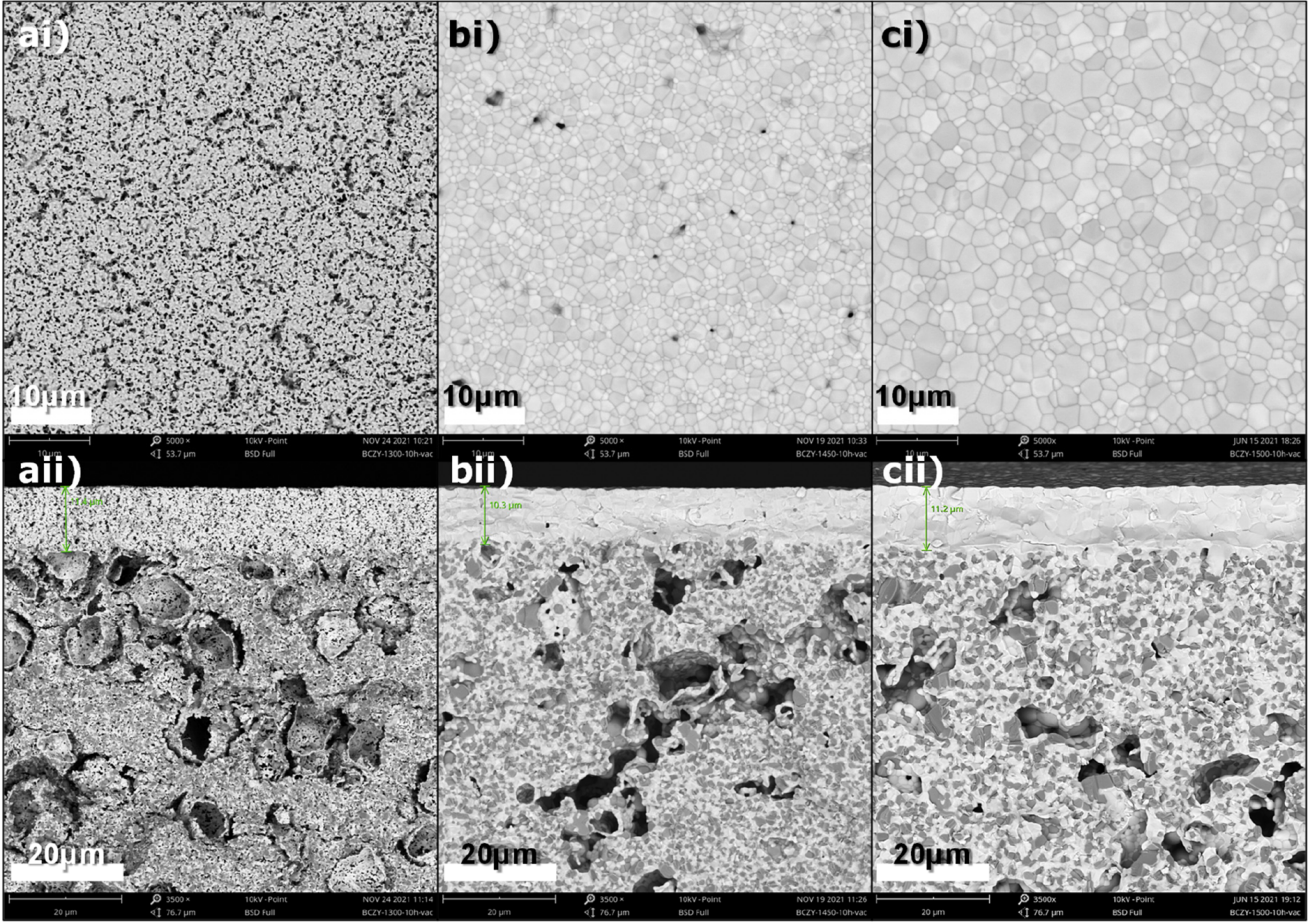
SEM images of the half-cells sintered for 10 h at a temperature of (a) 1300, (b) 1450, and (c) 1500 °C. The surface of the membrane is shown in ai–ci, and the cross-section of the half-cells with the membrane and the support electrode is shown in aii–cii.

The sinterability and microstructure of the membrane/electrode depend upon the sintering temperature and time, as reported previously.^40,42^ We tested three different coating techniques: spray, spin, and dip coating on our presintered supports (Figure S5). Membrane coatings deposited by spraying result in macrocracks and uneven layers, possibly due to the fast evaporation of volatiles and irregular deposition. Similarly, coatings formed via the spin drop coating method (Figure S5b) result in thicker layers but contain pinholes and pores. Sintering of these coatings was also unsuccessful, as many of the coatings became extremely brittle and shattered upon touch due to possible residual stresses. We observed the best results with vacuum-assisted dip coating and thus continued to optimize the process around this method. This technique enables the coating of a uniform and thin electrolyte layer by compensating for any surface irregularities. Moreover, it packs the material densely and locks within the pores that aids in densification without delamination or fracture upon sintering. With this technique, we also observed that the temperature plays a critical role in the densification of the electrolyte. For example, as shown in Figure 4ai-aii (surface and cross-section), the electrolyte is very porous at 1300 °C, so it cannot be used for practical applications. However, by increasing the sintering temperature from 1300 to 1450 °C (Figure 4bi, bii), a remarkable improvement in the densification is observed, but some pores are still present that can lead to gas crossover. Thus, when the sample is sintered at a higher temperature of 1500 °C, a completely dense layer is observed (Figure 4ci, cii and Figure S6).

**Figure 5:**
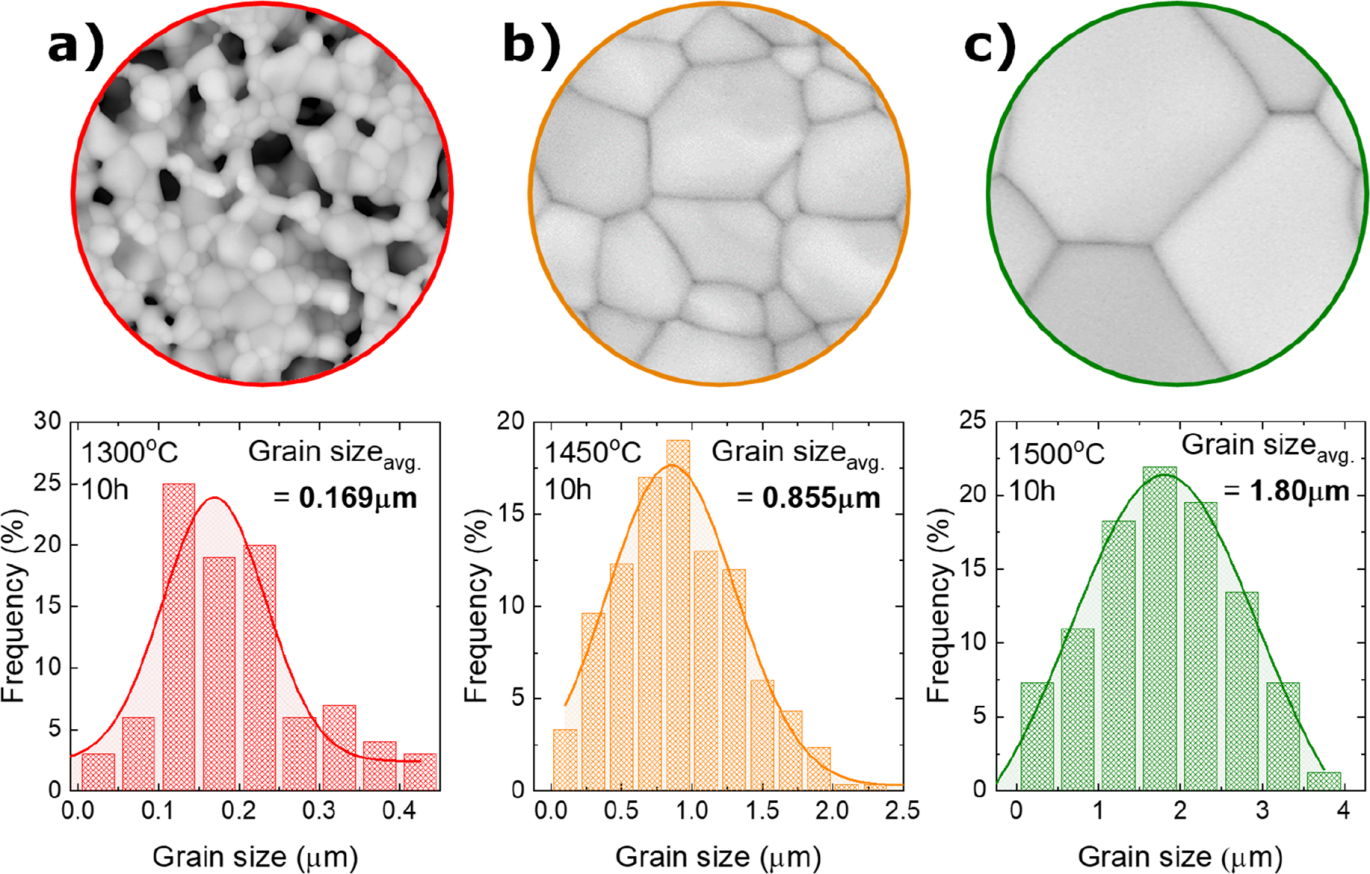
Influence of sintering temperature on the grain size of BCZY271 sintered for 10 h at (a) 1300, (b) 1450, and (c) 1500 °C. SEM images are of the same scale and magnification.

From Figure 4, it is evident that the temperature directly influences the microstructure and densification without the addition of any sintering aid. The average grain size varies from 0.17 to 1.8 μm as the sintering temperature is increased from 1300 to 1500 (Figure 5). The pore size in the electrolyte varies from 2 to 5 μm at 1300 °C to 0 μm with an increase in temperature to 1500 °C. Moreover, the overall half-cells show a shrinkage of 14% (1300 °C), 20% (1450 °C), and 23% (1500 °C) (Figure S7), which further assists in the densification of the membrane.

#### 3.2.2 Effect of Sintering Time

**Figure 6:**
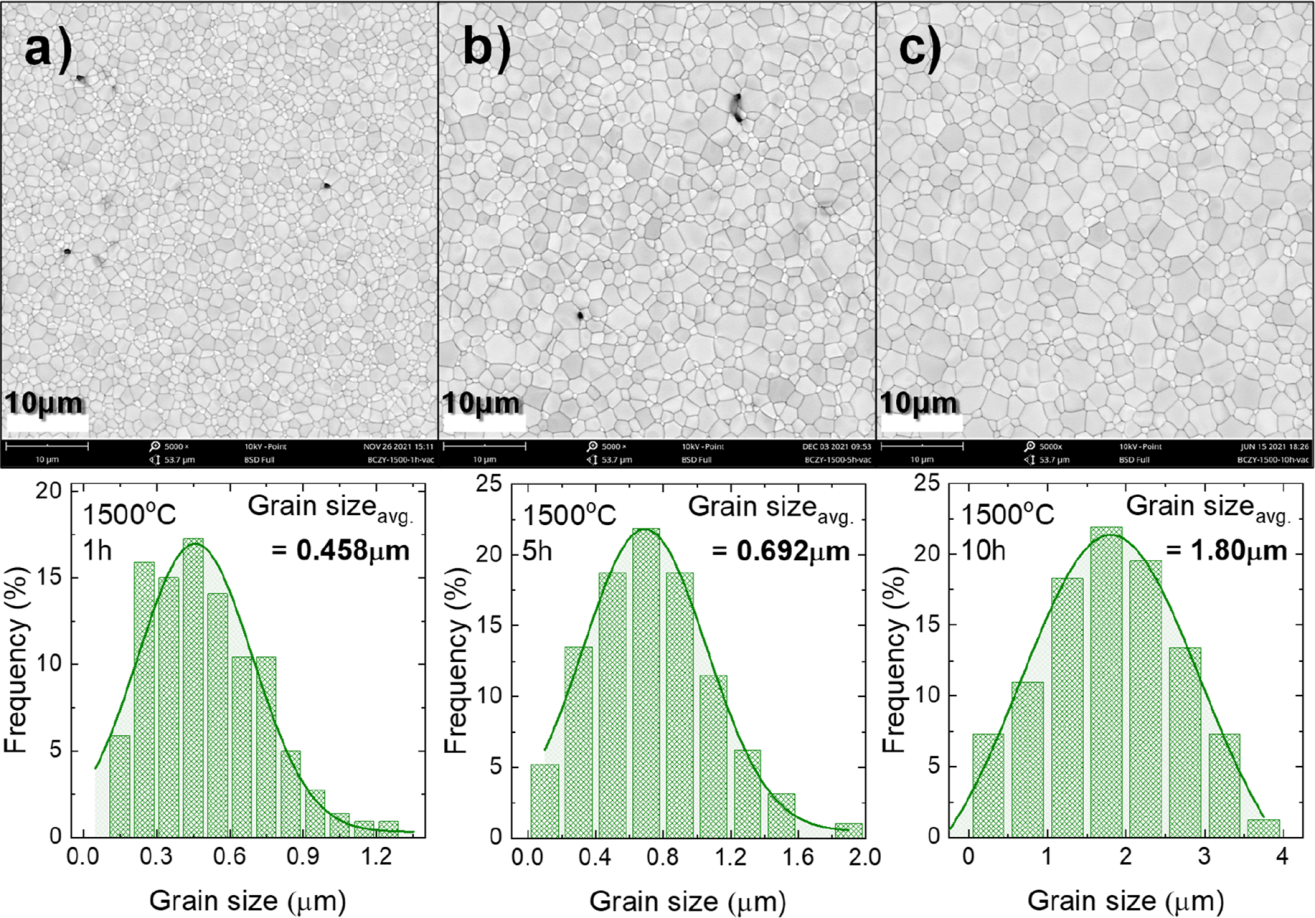
Effect of time duration on the grain growth of BCZY271 sintered at 1500 °C for (a) 1, (b) 5, and (c) 10 h.

After noticing the effects of temperature on the microstructure, we also observed the effect of varying time on the grain growth of the membrane at a temperature of 1500 °C, as shown in Figure 6. Increasing the sintering time not only leads to an increase in the grain size but also decreases the porosity/defects. For example, the number of pores/defects is practically eliminated after 10 h of sintering (Figure S8). Moreover, the grain size is increased from 0.46 to 1.8 μm as the sintering time is increased from 1 to 10 h (Figure 6), as also observed in ref (40). Based on the results obtained for varying sintering temperature and time, we selected a sintering temperature and time of 1500 °C and 10 h, respectively, for the fabrication of our cells, as the electrolyte was found to be completely dense (to prevent gas crossover), and the grain size was large, which essentially reduces the blocking effect. However, since 1500 °C is quite a high temperature, we also analyzed the stability of the material in the following section by performing XRD for the surface of the electrolyte sintered at 1500 °C for 1–10 h.

#### 3.2.3 Structure and Thermodynamic Stability of the Electrolyte Layer

**Figure 7:**
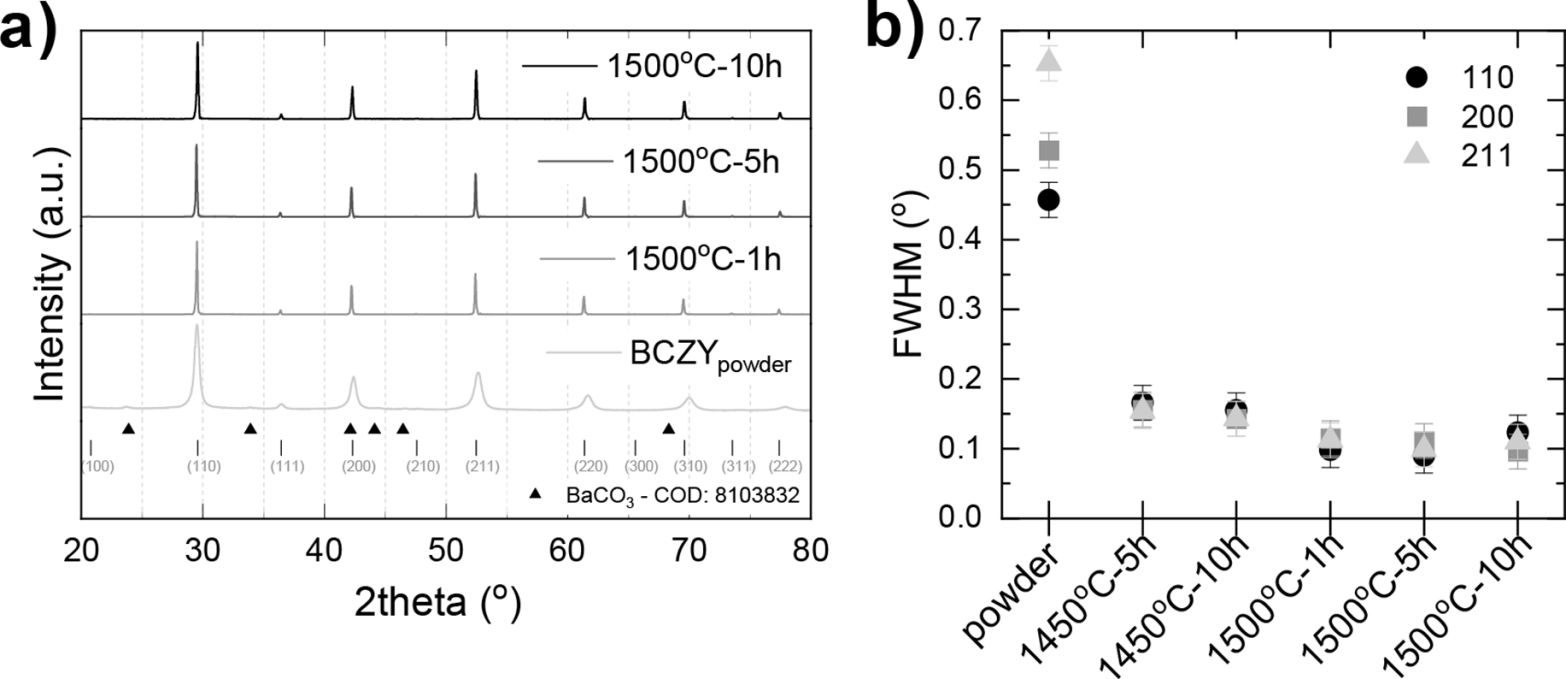
(a) XRD patterns for BCZY271 sintered at 1500 °C for 1, 5, and 10 h compared with the commercial powder, (b) estimation of the full width half-maximum for the 110, 200, and 211 peaks.

X-ray diffraction (XRD) analysis was performed to check the purity and crystal structure of the starting material as well as the dense electrolyte after sintering at 1500 °C for 10 h. The commercial powder BCZY271 contains slight traces of barium carbonate (Figure 7a). A slight peak shift upon sintering is observed, which is indicative of change in the lattice parameter. This can be attributed to three effects: (i) the redistribution of surface barium, which is available as BaCO_3_ in the perovskite powder, to an extended crystal structure, and as a consequence, the XRD pattern is shifted toward lower 2θ in comparison to the powder; (ii) the thermal reduction and oxygen loss associated with high temperature treatment; and (iii) uniform strains causing the peak shifting. At 1500 °C, the cubic crystal structure is observed to be retained, and the lattice parameter of 4.2682 Å is found to lie within close range of the pseudocubic lattice parameter reported in the literature for similar compositions of BCZY.^27,40,67^ After sintering, the spectra show no impurity regardless of the sintering time, which is otherwise often observed in simultaneous SSRS methodology. From Figure 7b, the observed decrease in the fwhm with temperature can be related to the increase in crystallite size that shows tremendous reordering and growth, the average crystallite size from ∼20 nm to ∼100 nm or the associated straining effect upon densification.

Elemental spot analysis was also performed on the surface of the sintered membrane to determine the stoichiometry upon sintering. The ABO_3−δ_ quantitative ratio was found to be consistent with the stoichiometry of the BCZY271 (BaCe_0.2_Zr_0.7_Y_0.1_O_3−δ_) precursor used for deposition (Figure S9). This indicates that 5–10 h of sintering at 1500 °C is sufficient, but to achieve a dense and defect-free layer as depicted from the SEM, a sintering time of 10 h is suggested. Based on these results, we continued to use a sintering temperature and time of 1500 °C and 10 h, respectively, for the fabrication of half-cells since the electrolyte prepared under these conditions was found to be dense and defect free.

### 3.3 P-SOC Characteristics and Performance Evaluation

#### 3.3.1 Microstructure

**Figure 8:**
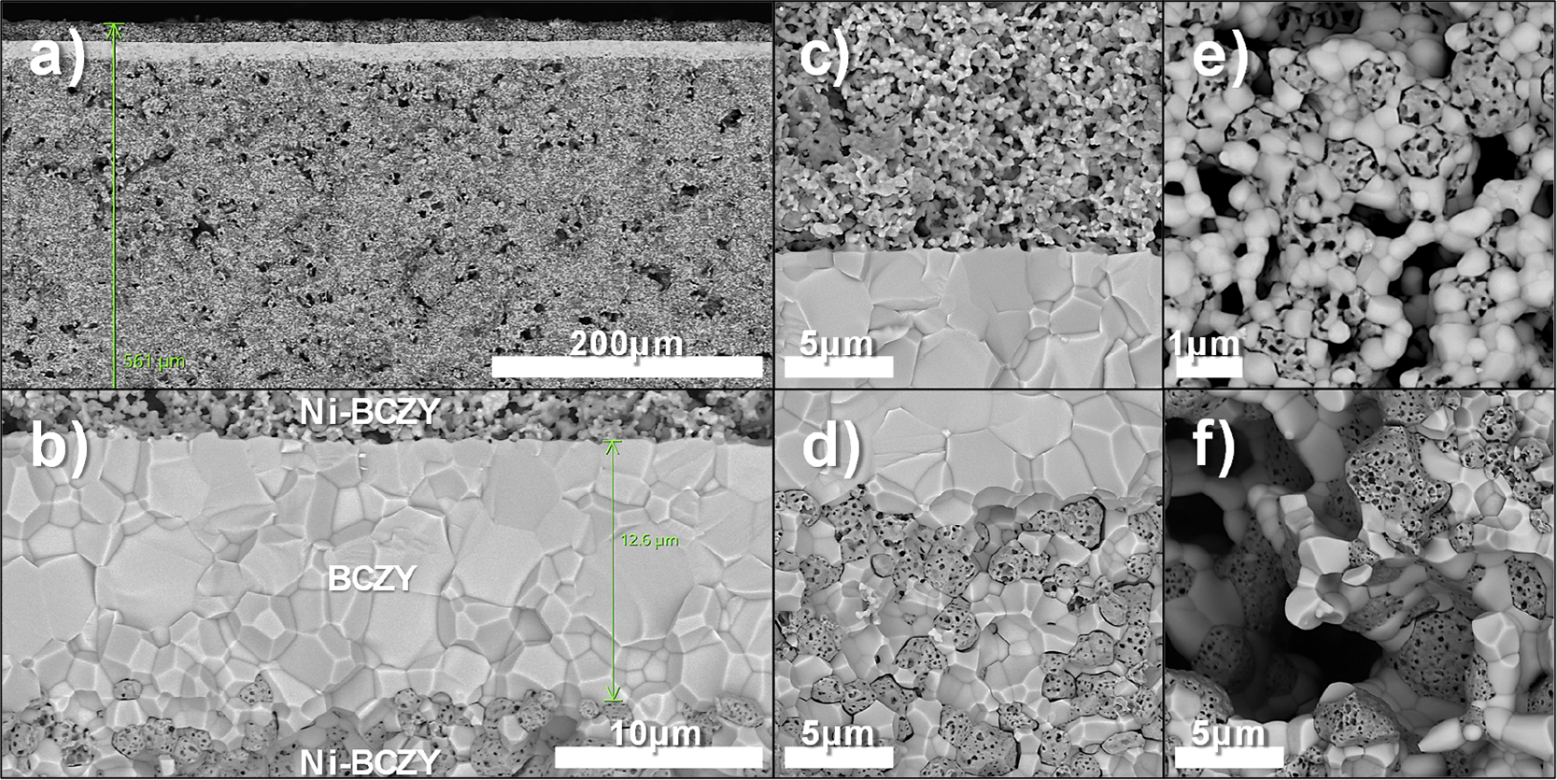
Fractured cross-section SEM image of a protonic ceramic cell: (a) the printed electrode–electrolyte-support electrode, (b) magnified image of the BCZY271 electrolyte layer, (c) printed electrode–electrolyte interface, (d) electrolyte–support electrode interface, (e) magnified image of the printed electrode, and (f) magnified image of the support electrode.

As mentioned in the experimental section, an asymmetrical single cell was fabricated by screen printing a NiO-BCZY layer (1400 °C - 4 h) on top of the half-cell (Figure S1). The complete cell consists of a porous support electrode (NiO-BCZY, average thickness 550 μm), a dense electrolyte (BCZY, thickness 12.6 μm), and a printed electrode (NiO-BCZY) with an average thickness of 15 μm (Figure 8).

The average grain size of NiO and BCZY in the screen-printed electrode is below 1 μm (Figure 8e). We initially chose to sinter this electrode at 1150 and also 1300 °C, but since the electrolyte surface is relatively smooth, the poor adhesion resulted in interfacial delamination. Thus, we chose to sinter at 1400 °C which retains the adhesion even after long-term aging test as depicted in Figure S13. Prior to electrochemical evaluation, the cell was reduced in hydrogen. As a result, a drastic change in the nickel morphology of the support electrode is observed in addition to the macropores/micropores of the electrode (Figure S4). This change in the microstructure leads to the generation of spongy nickel (Figure 8f), which increases the porosity from 4% to 23% (Table 2). In addition, an elemental map analysis was also performed for a completely reduced cell. A homogeneous distribution of various elements was observed in the respective electrode and electrolyte layers (Figure S10). No nickel presence was observed inside the electrolyte layer, which fairly excludes the possibility of nickel diffusion as a consequence of elevated temperature. However, the high intensity of ceria is observed in both electrodes. If ceria intensities were present only in the support electrode and electrolyte, we could attribute them to the phase separation caused by high temperature (1500 °C) and long-term treatments (10 h). But since the highlights are also observable at the same locations as of nickel in the screen printed electrode which is sintered at 1400 °C and 4 h, we exclude the possibility of phase separation due to high temperature treatment. Instead, we believe the emission energies of ceria Ma (0.88 keV) and Mb (0.90 keV) to coincide at the tail of nickel peak at La1 (0.85 keV) and Lb1 (0.87 keV) during the mapping, and thus the software registers the nickel peak intensities as the presence of ceria also. Moreover, since the scan is performed on a fractured surface and not polished one, some surfaces are closer to or protruding out of the scan plane leading to high contrasts, gives the impression of inhomogeneous distribution.

#### 3.3.2 Electrochemical Evaluation

**Figure 9:**
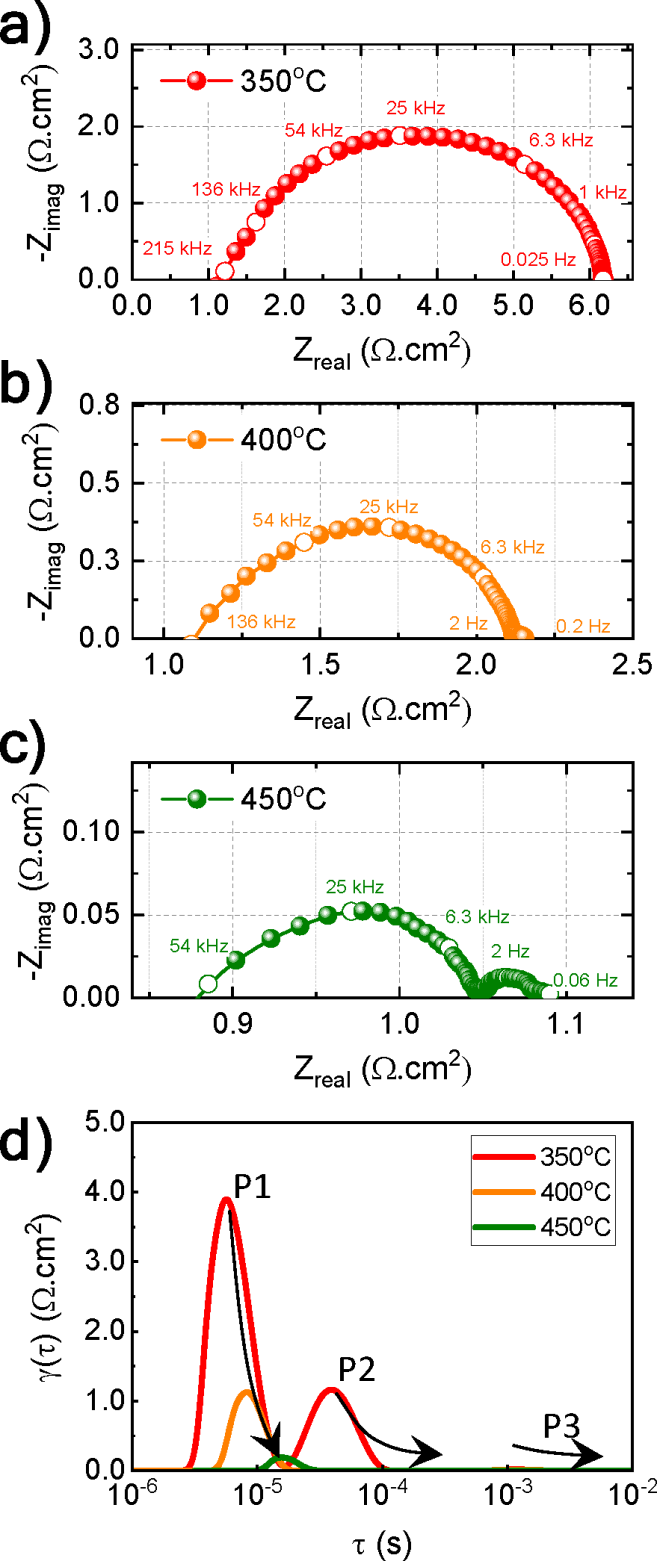
Nyquist plots for the EIS measurements performed near open circuit potential at (a) 350, (b) 400, and (c) 450 °C under humidified hydrogen conditions (10 vol % H_2_) in helium at the negatrode | humidified hydrogen (60 vol % H_2_) in helium at the positrode. (d) Distribution of relaxation time (DRT) analysis of the P-SOC using discretization parameter, lambda order: 1 × 10^–4^. P1, P2, and P3 are associated with the charge transfer reaction, formation of nickel–hydrogen bonding, and hydrogen adsorption and desorption processes, respectively.

EIS was performed at OCV to probe into the initial ohmic resistances of our P-SOCs, separate the resistance of the membrane from the electrodes, and determine whether the positrode is suitably porous for gas diffusion. The ohmic resistance of the P-SOC, represented as *R*_o_, includes contributions from the bulk proton transport, electrode thickness, electrical contact, and wire resistances. *R*_o_ is obtained from the high frequency intercept with the real axis, whereas the electrode polarization resistance, *R*_P_ (charge transfer, adsorption/desorption), is estimated from the width of the depressed arcs, i.e., the difference between the high- and low-frequency intercepts. Resistance due to gas diffusion is depicted from the low frequency ending. Figure 9a–c shows the electrochemical impedance spectra represented by Nyquist plots measured at 350, 400, and 450 °C under open-circuit and humidified hydrogen conditions. To further distinguish the electrode processes, we determined the distribution of relaxation times (DRT) by using MIT free-ware DRTtools and *MATLAB* with an optimization toolbox. The computation was based on Tikhonov regularization using the fit parameters reported in ref (68). Figure 9d shows the distribution of the relaxation time (DRT) for the same, which can be used to deconvolute and identify the processes involved during operation.

The area specific ohmic resistances (ASR) and *R*_o_ were determined to be 1.15, 1.09, and 0.87 Ω cm^2^ at 350, 400, and 450 °C, respectively. The decrease in *R*_o_ is attributed to an increase in proton mobility with increasing temperature. The *R*_P_ at 350 °C is 5.02 Ω cm^2^, which is 4.4 times higher than *R*_o_ and can be associated with the activity of nickel for hydrogen oxidation (HOR) and evolution (HER) reactions. However, *R*_P_ is decreased to 1.06 Ω cm^2^ (0.97 times) at 400 °C and 0.22 Ω cm^2^ (0.25 times) at 450 °C compared to the bulk resistance, *R*_o_. The data plots in the Figure 9a–c are inclusive of the inductive responses from the wires, membrane-electrode assembly and the measurement instrument. Frequency range ascribed to the grain and grain boundary processes may appear below the real axis and into the positive imaginary region due to the inductance. The disappearance or lack of resolution of the grain boundary arcs in this work is attributed to the same phenomena as reported in ref (69).

**Figure 10:**
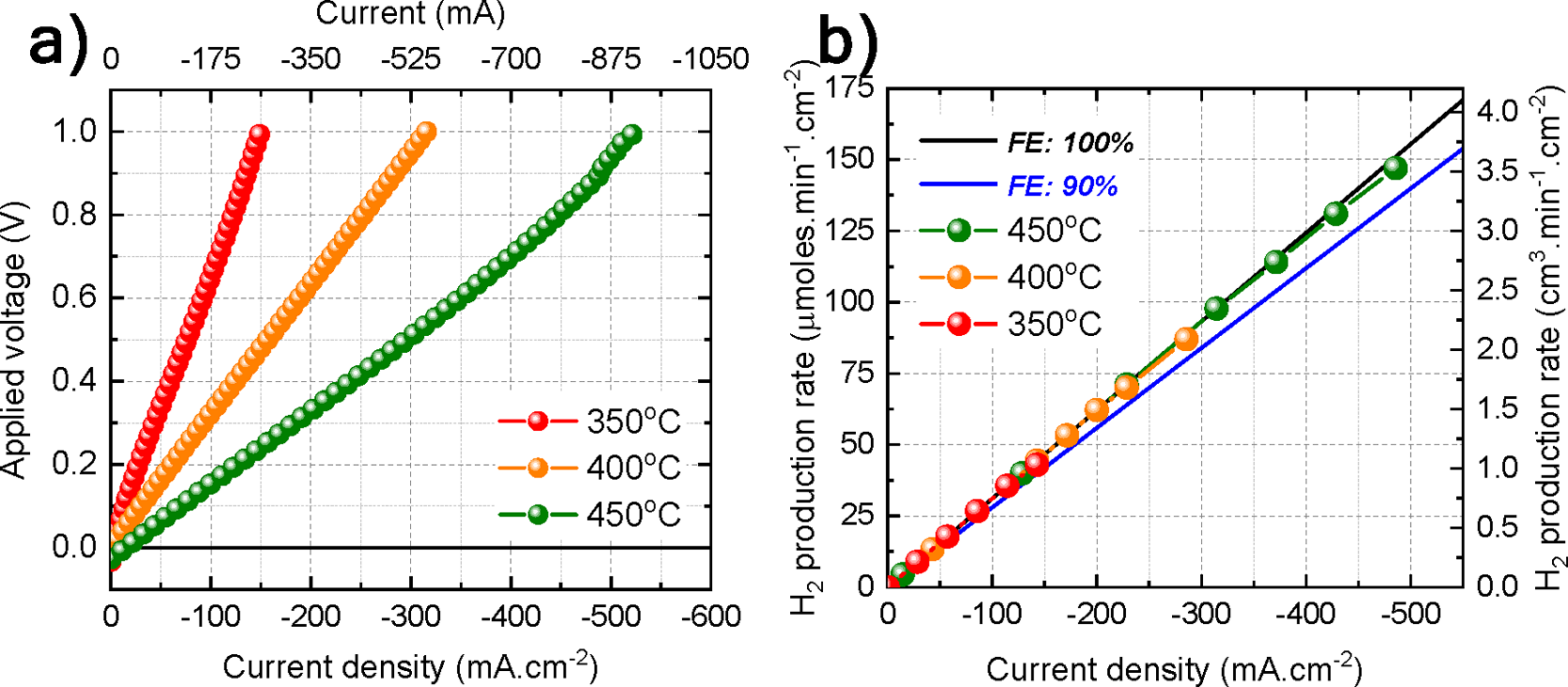
(a) *I*–*V* polarization characteristics and (b) faradaic efficiency between 350 and 450 °C for the P-SOC evaluated as a hydrogen pump under humidified helium at the negatrode | humidified hydrogen (60 vol % H_2_) in helium at the positrode.

As depicted in Figure 9d, at least three processes can be identified to occur at a certain temperature, labeled P1, P2, and P3. On the basis of NiO-BCZY reduction temperature and the evaluation of activation energies corresponding to the DRT peaks reported in ref (15), P1 is associated with the charge transfer reaction between Ni and the BCZY interface, P2 is related to the formation of nickel-H solutions (α-phase), and P3, which is slightly visible at 350 °C, is related to the hydrogen adsorption and desorption processes on the nickel surface. From an estimate of the time constant for each peak P1 at 350, 400, and 450 °C, charge transfer is determined to occur at approximately 200, 140, and 70 kHz. The formation of nickel-H solution is observed to occur only at 350 °C and approximately 5 kHz. The P3 process is observed to contribute negligibly at 350 °C and is nonexistent at higher temperatures. We can see that with an increase in the temperature, P1 decreases strongly, while P2 and P3 are nearly diminished, indicating that the device performance is governed by charge transfer reactions only. The reliability of the measurement is presented in Figure S11 with suitable equivalent circuits comprising of inductor (L), resistor (R), and constant-phase-element (CPE) arrangements are suggested in the insets. Since the residuals for the linear KK test fall below 1%, the impedance data demonstrate the true nature of the device under equilibrium. The performance of the P-SOC as a hydrogen pump or separator when tested between 350 and 450 °C is reflected by the *I*–*V* characteristics shown in Figure 10a.

As expected, the current increases linearly with applied potential and increasing temperature. For instance, at an applied potential of 1 V, the current density increases from 150 to 525 mA/cm^2^ as the temperature is increased from 350 to 450 °C. In addition, the faradaic efficiency at each temperature was measured to evaluate the protonic current, as plotted in Figure 10b. Regardless of the temperature and current, the faradaic efficiency is above 98%, which is in fair agreement with the theoretical calculation. As a comparison, electrochemical pump constructed with a highly conductive membrane material like BaCe_0.7_Zr_0.1_Y_0.2_O_3-δ_ (BCZY712) allows equivalent fluxes at a lower required potential compared to ours. But based on the architecture, membrane-electrode construction and faradaic efficiency, our P-SOC outperforms at any applied potential and temperature.^15,16^ FE close to theoretical value indicates a membrane free of short circuits and electronic conductivity. Even when not used as a sintering aid, nickel is likely to diffuse at high temperature from the support to the electrolyte during solid state reactive sintering (SSRS) of the precursors where it forms intermediate phases and settles at the grain boundary; causes electronic conductivity leading to short circuiting and low FE. Our presintering of the support followed by the application of a perovskite phase-ready thin layer decreases the likelihood of nickel diffusion into the electrolyte.

**Figure 11:**
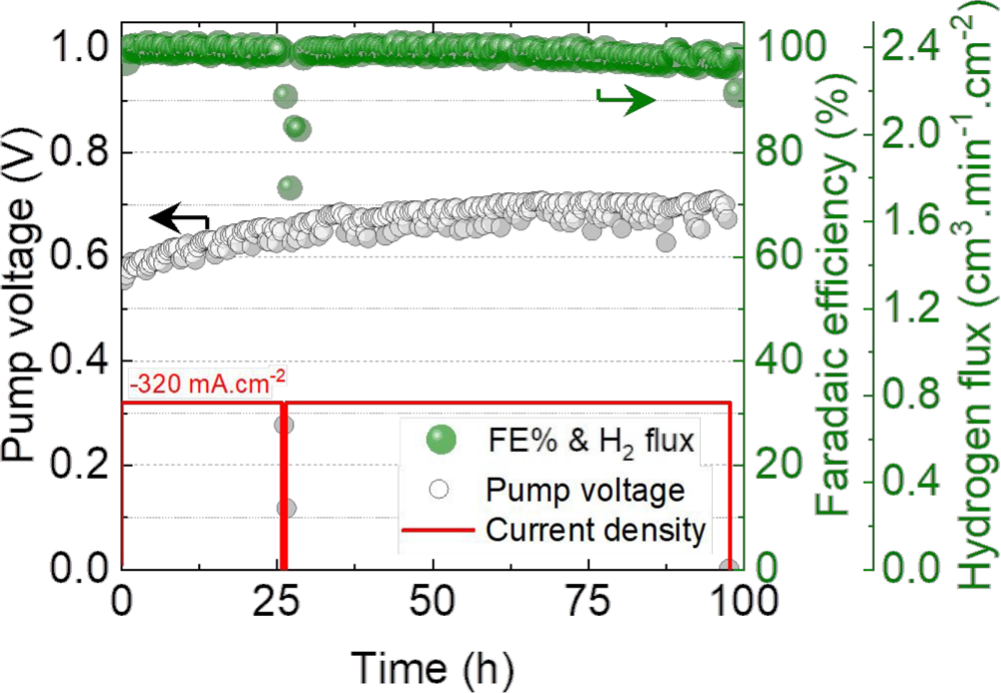
Aging behavior of the P-SOC operated for 100 h at 320 mA cm^–2^ at 450 °C under humidified helium at the negatrode | humidified hydrogen (60 vol % H_2_) in helium at the positrode.

Following the *I*–*V* measurements, the cell was aged for 100 h at 320 mA cm^–2^ at 450 °C. As shown in Figure 11, the required voltage shows an initial value of approximately 560 mV, which is followed by an increase of 120 mV until 30 h. The calculated degradation rate is approximately 4 millivolts per hour for the initial 30 h. After the initial aging, the potential appears to stabilize afterward. This initial increase in the required overpotential can be the result of reordering or shifting of the triple phase boundary (TPB), mainly due to nickel shape transformation, migration, or loss of contact with the BCZY at the interface, which is a typical first observation in nickel-based cermet electrodes.^70^

The EIS and *I*–*V* polarization curves measured before and after the aging test are presented in Figure S12. As clearly visible, there is a slight increase in the ohmic and overall polarization resistance, which is reflected in the form of an increased required potential to drive the protons across the membrane. We ascribe this increase in *R*_o_ and *R*_P_ to the generation of microporosity within the nickel particles, as it is leading to the decrease in interfacial contact points with the BCZY grains at the membrane interface, or with the adjoining nickel within the electrodes. We confirmed this as the post-test SEM analysis (Figure S13) reveals no signs of electrode delamination or pronounced nickel migration away from the interface and thus attribute microporosity to be the only cause of the initial degradation.

## 4 Conclusion

In this work, we have successfully demonstrated the development of a P-SOC with a defect-free and dense electrolyte (BCZY271) prepared by a vacuum-assisted dip coating technique on a porous nickel BCZY support. A detailed description of each processing step, starting from the fabrication of the support electrode to the electrolyte coating and sintering, is presented. A half P-SOC was converted to a full cell by screen printing of a NiO-BCZY electrode. The complete Ni-BCZY (∼550 μm)/BCZY (∼12 μm)/Ni-BCZY (∼15 μm) P-SOC was evaluated as a hydrogen pump in the temperature range of 350 and 450 °C, reaching a maximum current density of 525 mA cm^–2^ at 1 V and 450 °C. The faradaic efficiency is found to remain above 98% over a broad range of current densities. The short-term aging shows insignificant degradation after 30 h and remains stable for another 70 h until the end of the test.

The optimized conditions for the support electrode with a planar architecture are 10 wt % dextrin type II pore former, 150 MPa for the compaction stress and a presintering step of 1300 °C. We conclude this information from the fact that the 0.56 mm thick supports are quite strong and remain intact during coating and handling. Despite densification at 1500 °C for 10 h, the supports remain suitably porous and strong upon reduction. This is also reflected in the superior *I*–*V* characteristics and the EIS analysis, where the major losses are found to be due to only bulk and charge transfer resistance, with no sign of diffusion resistance due to electrode densification observed.

With our coating technique, we can achieve a higher level of membrane densification and suitable grain growth without the use of sintering additives. We attribute this to the unique characteristics of the deposition technique, i.e., material embedding and creation of a rooted structure within the support electrode that enables better adherence and densification compared to other coating techniques. We suggest sintering samples at 1500 °C for 10 h, as an electrolyte prepared under these conditions is found to be considerably dense without material decomposition or warping, which is further supported by SEM, XRD, elemental spot, and faradaic measurement analyses.

These promising results suggest that P-SOC half-cells prepared using our fabrication technique and parameters can be upgraded and implemented in various applications, such as fuel cells, water, and/or carbon dioxide electrolysis, and for the production of synthetic fuels or ammonia with the application of a suitable electrode.

## Supporting Information Available

Additional information for the optical micrographs of the sintered half-cells, P-SOC current collector application, sealing with glass and the installation steps toward evaluation, initial reduction of the P-SOC after installation, the results for the tested coating types, plot on densification of the membrane and shrinking of the electrode with temperature, estimation of electrolyte defects/pores vs time from SEM imaging, elemental spot analysis of the sintered membrane surface, elemental map analysis of the complete cell, Kramers–Kronig residuals for the EIS spectra with suggested equivalent circuits, P-SOC aging *I*–*V* and EIS plots, and postaging test SEM micrograph (PDF)

## Notes

Raw data that were used for the plots within this paper and the accompanying Supporting Information are available to download from https://doi.org/10.5281/zenodo.6778474.
