# Supplementary material for: Development of Electrode-Supported Proton Conducting Solid Oxide Cells and their Evaluation as Electrochemical Hydrogen Pumps
Source: ACS Appl Mater Interfaces. 2022 Aug 18;14(34):38938–51. doi: 10.1021/acsami.2c11779 (PMC9472216; doi:10.1021/acsami.2c11779)
Supplement: Supplementary file 1 — am2c11779_si_001.pdf [file am2c11779_si_001.pdf]

Supporting Information

Development of Electrode Supported Proton Conducting Solid Oxide Cells and their Evaluation as Electrochemical Hydrogen Pumps

Usman Mushtaq[a,b,], Stefan Welzel[a], Rakesh K. Sharma[a], M.C.M. van de Sanden[a,c], Mihalis N. Tsampas[a,*]*

a) Dutch Institute For Fundamental Energy Research (DIFFER), 5612AJ Eindhoven, The Netherlands.

b) Department of Chemical Engineering and Chemistry, Eindhoven University of Technology, 5600 MB Eindhoven, The Netherlands.

c) Department of Applied Physics, Eindhoven University of Technology, 5600 MB Eindhoven, The Netherlands.

[*] corresponding authors: U.M: u.mushtaq@differ.nl, M.N.T: m.tsampas@differ.nl

Sintered half cells optical micrograph

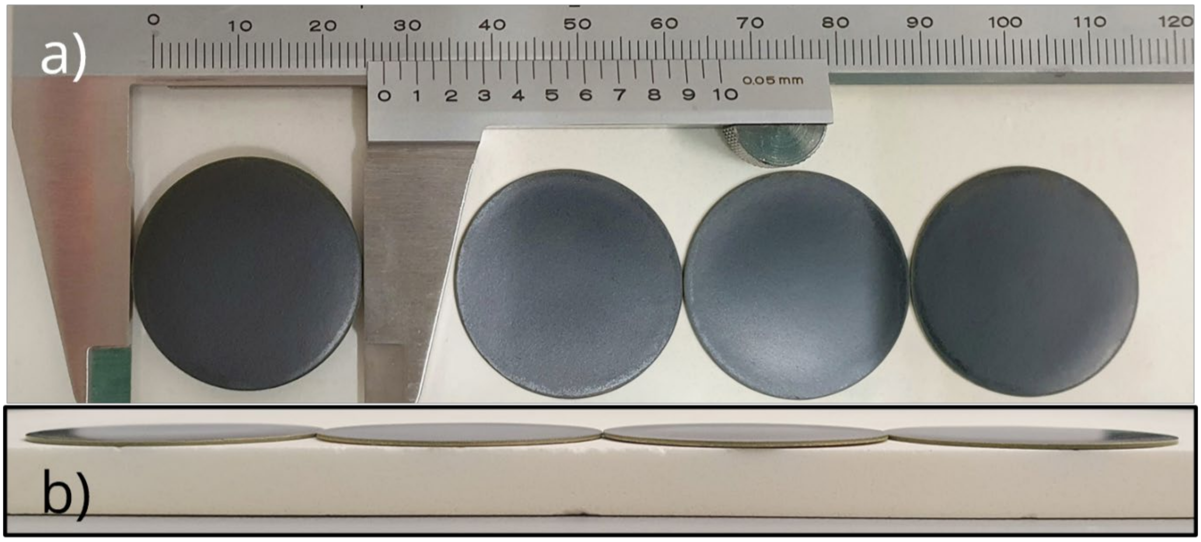

Figure S1: Optical image of the sintered half cells, a) top view with dimension and b) side view to indicate the flatness of the half-cell upon sintering at 1500 °C and 10 h.

P-SOC sealing and installation steps

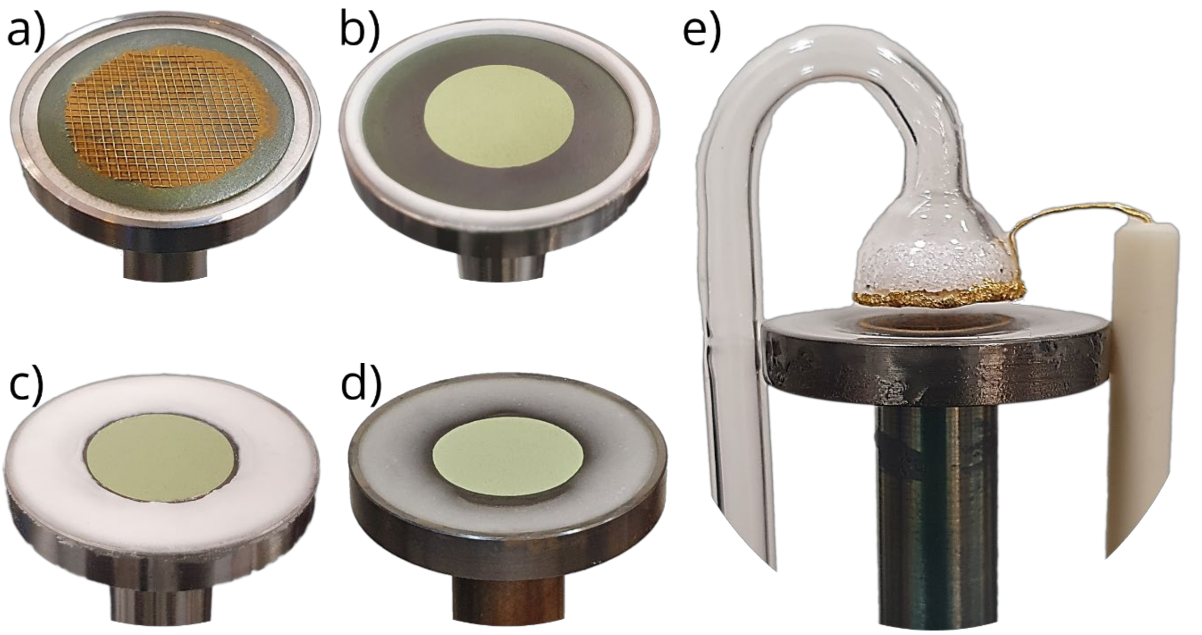

Figure S2: Sealing and installation methodology of the P-SOC. a) application of gold mesh and paste on the support electrode, b) sealing on the side of the support with glass paste, c) application of glass over the electrolyte, d) glass after curing at 700 ºC for 4 h and e) installation of the gold mesh and lead wires over the printed electrode under the gas diffusion apparatus.

Electrode reduction of NiO into Ni metal

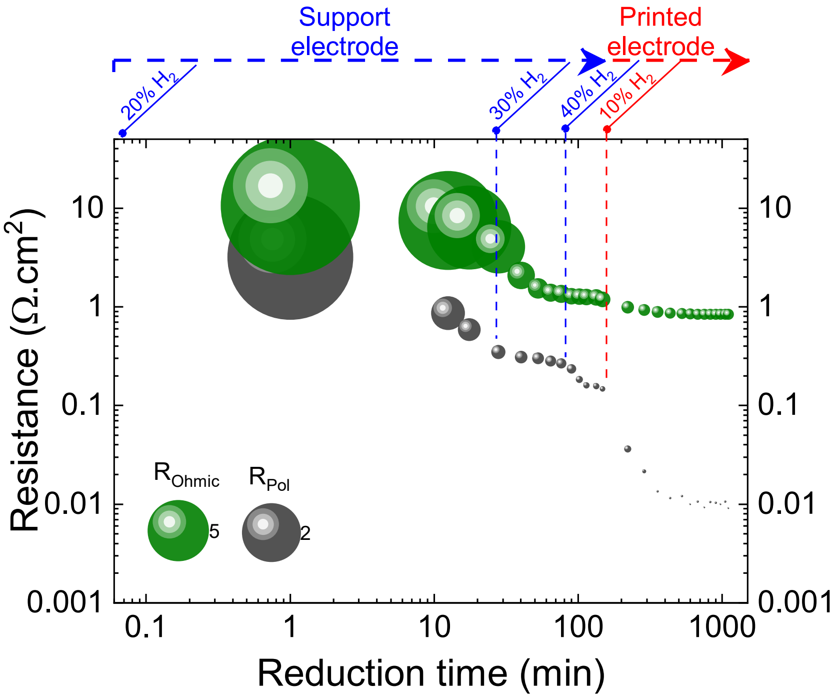

Figure S3: Variation of ohmic and polarization resistances with reduction and gas composition of the support (first in sequence) and printed electrode at 450 °C.

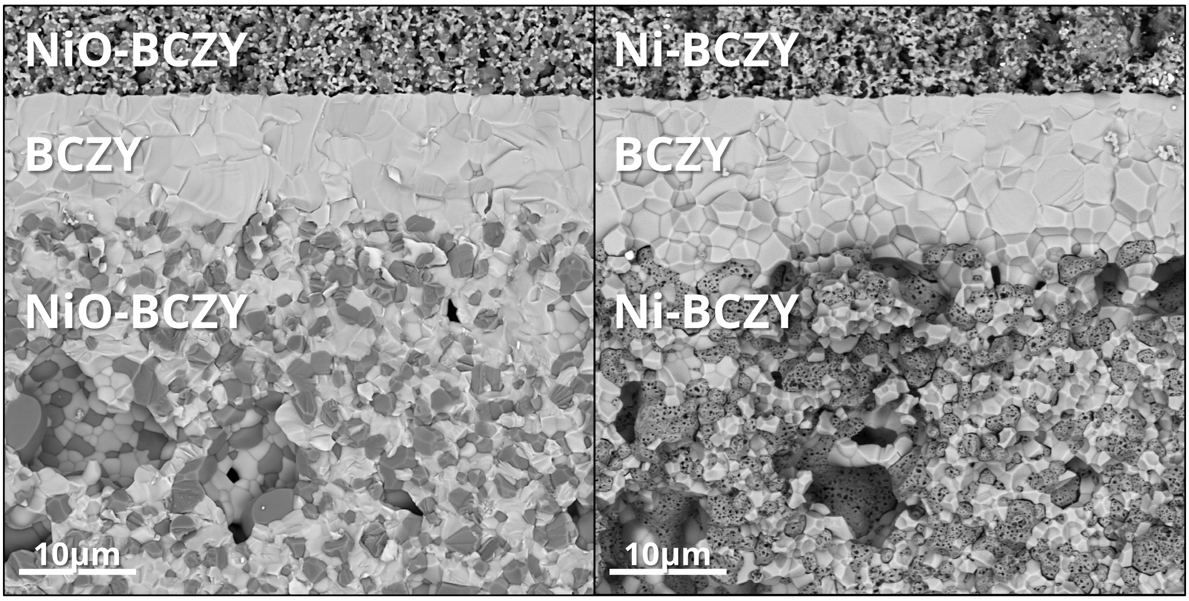

Unreduced Nickel Reduced Nickel

Figure S4: Fractured cross-section SEM images of the P-SOC before and after reduction process. Micropores are generated upon the reduction of nickel oxide to nickel. Images are from two different batch cells.

Results from tested coating types

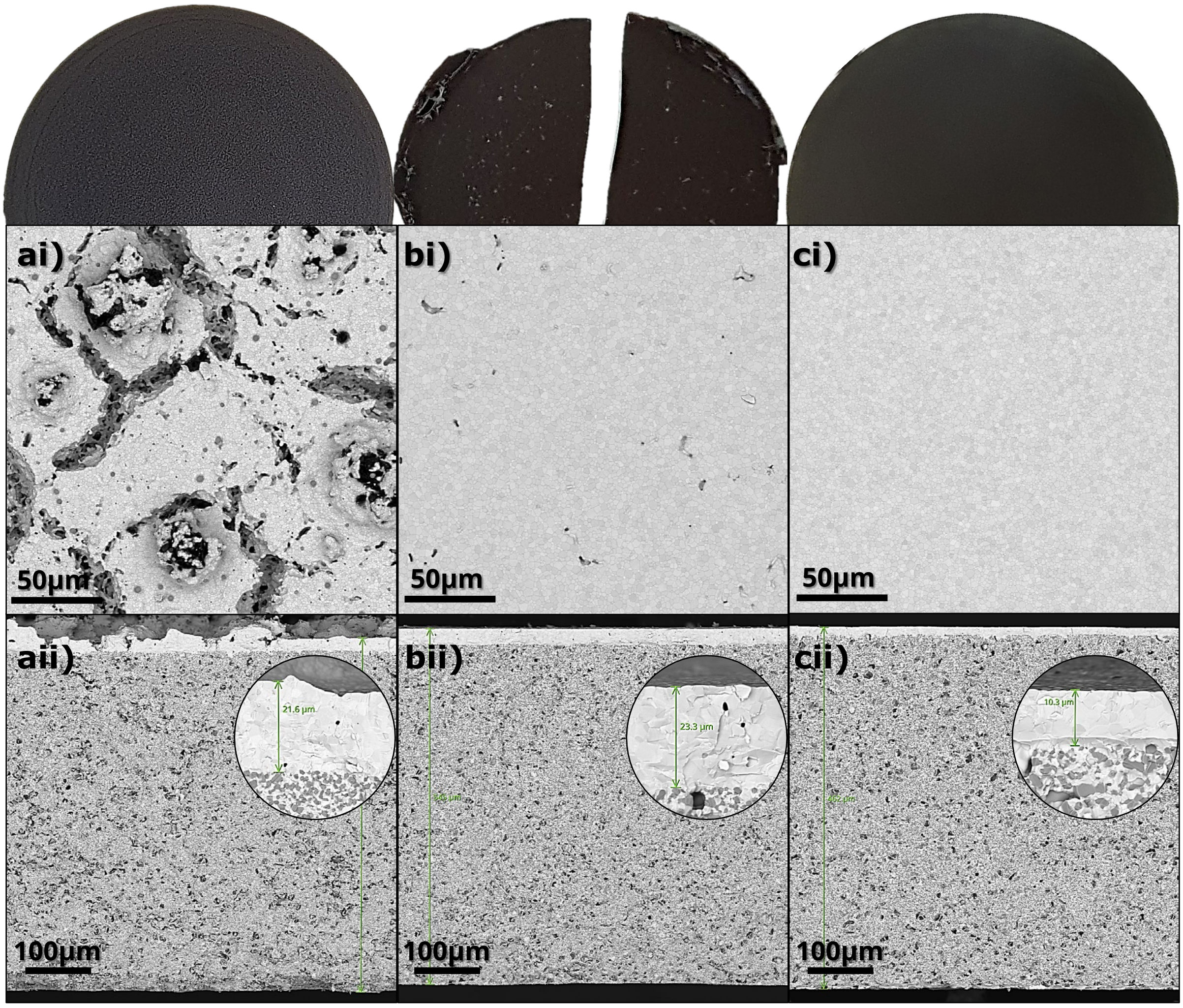

Figure S5: Physical characteristics of the three types of conventional coating methods employed to deposit layer of BCZY271 on the support electrode as a) spray-spin coating, b) drop-spin coating and c) vacuum assisted dip coating. On the top are the optical micrographs of the sintered half-cells at 1500 ºC for 10h. Surface of the coated layers is shown in ai-ci, whereas the cross-section is shown in aii-cii with an insert for higher magnification.

Densification of membrane surface with temperature

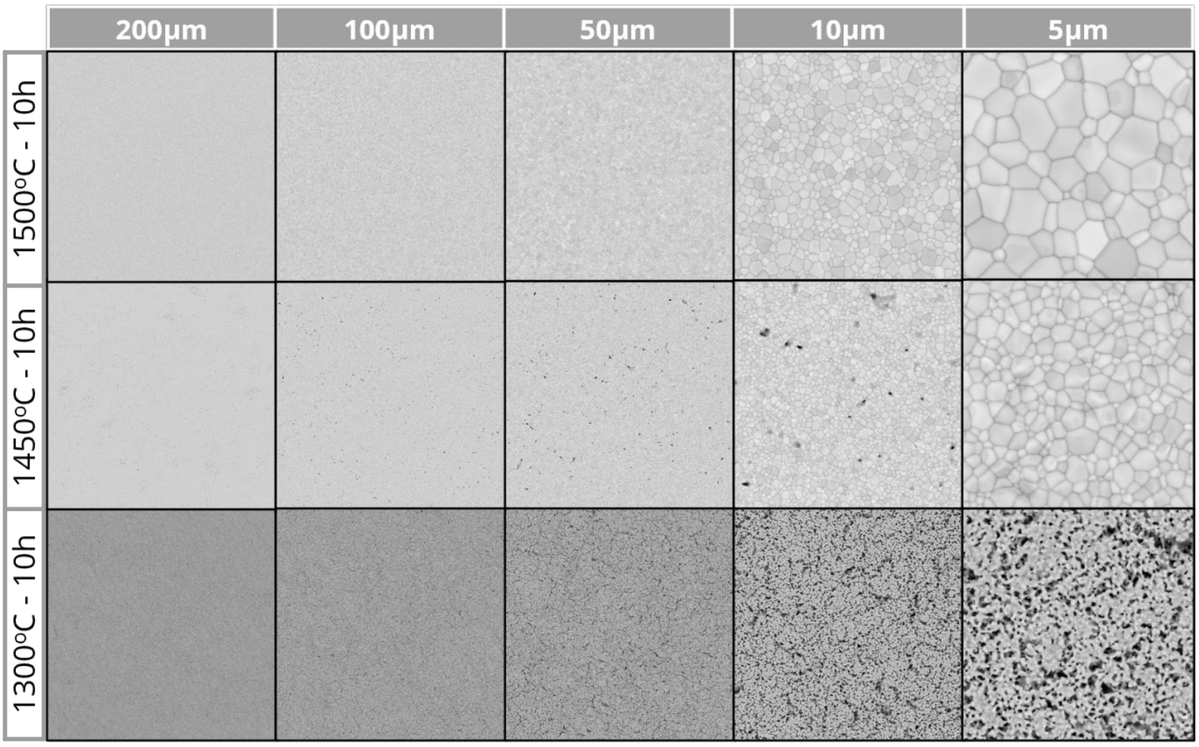

Figure S6: SEM overview of the membrane surface at various magnification levels and sintering temperatures between 1300 to 1500 ºC for 10 h.

Shrinkage of half cells with sintering temperature

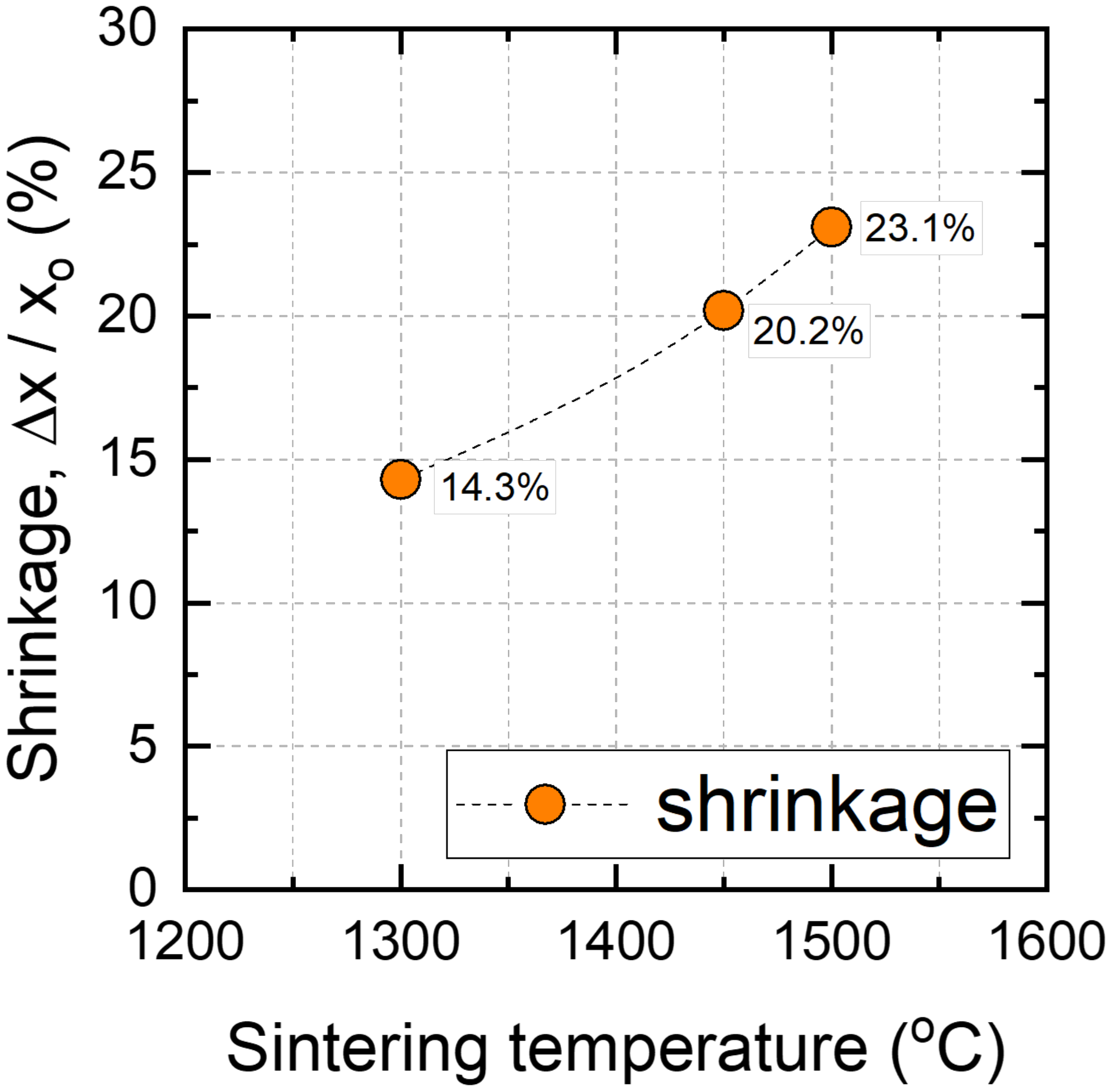

Figure S7: Overall geometric shrinkage of the sintered half cells at various temperatures. Higher shrinkage is also found to assist densification of the coated electrolyte layer.

Estimation method of defects and pores in electrolyte for various sintering time.

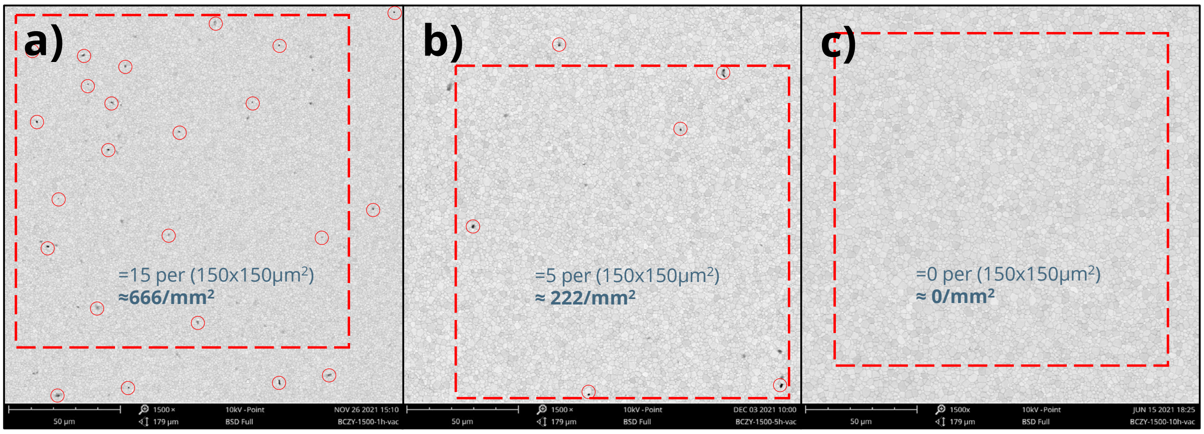

Figure S8: Representation and estimation of the defects present for various time duration of a) 1 h, b) 5 h, and c) 10 h sintering at 1500 °C.

Elemental spot analysis of the membrane surface after sintering.

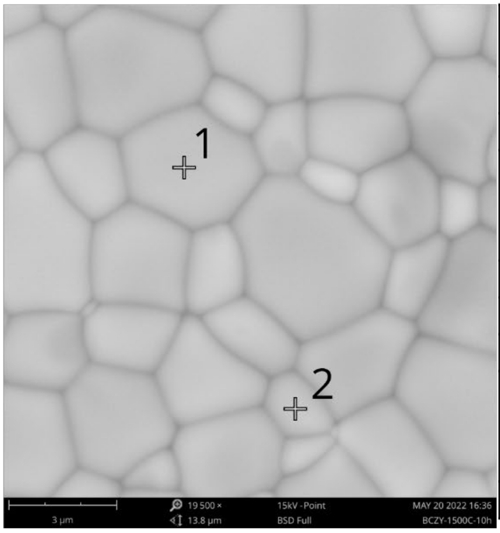

Element	Stoichiometry	Atomic concentration	Spot ratio	$ABO_{3-\delta}$
Spot 1				
Ba	**1**	15.27		1.00
Ce	**0.2**	2.91	0.19	
Zr	**0.7**	11.1	0.71	1.03
Y	**0.1**	1.71	0.11	
O	**3**	69.01		2.23
Spot 2				
Ba	**1**	16.2		1.00
Ce	**0.2**	2.94	0.18	
Zr	**0.7**	11.71	0.72	1.00
Y	**0.1**	1.53	0.09	
O	**3**	67.62		2.09

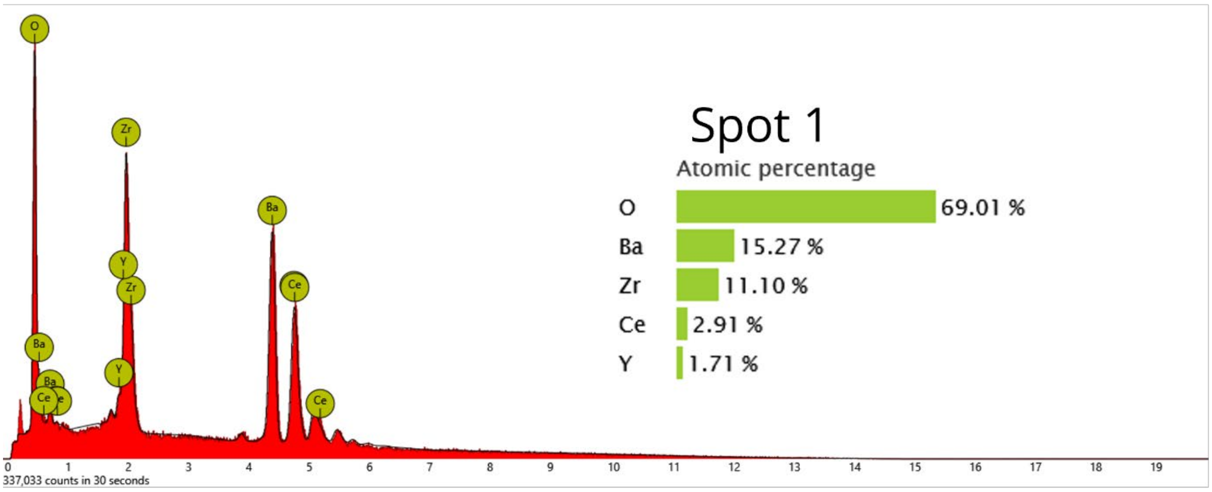

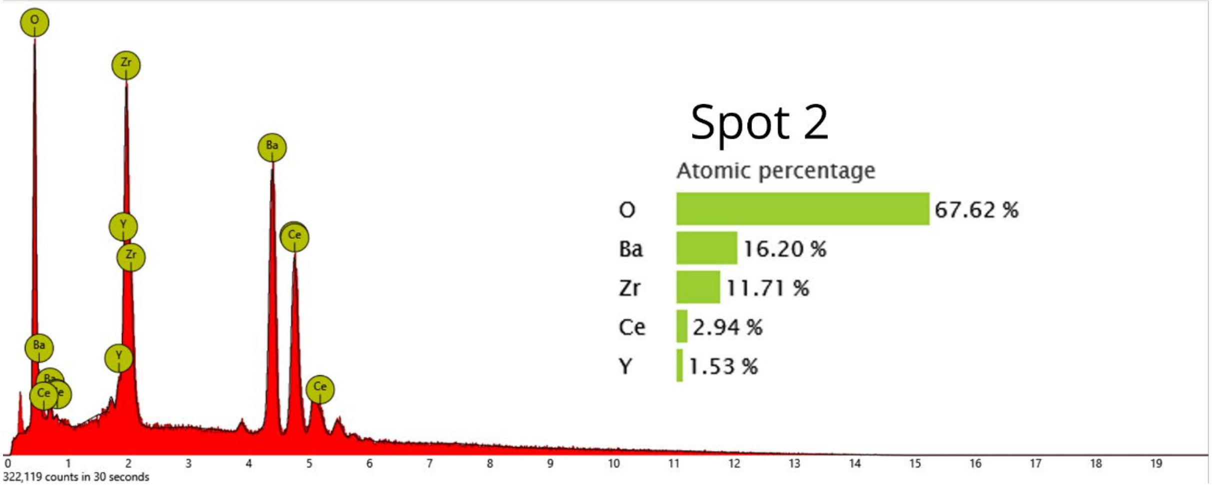

Figure S9: Elemental spot analysis (atomic percentage of the constituent elements) of the membrane surface sintered at 1500 °C and 10h. Carbon peaks are neglected due to background from the carbon tape.

Elemental map analysis of a reduced P-SOC.

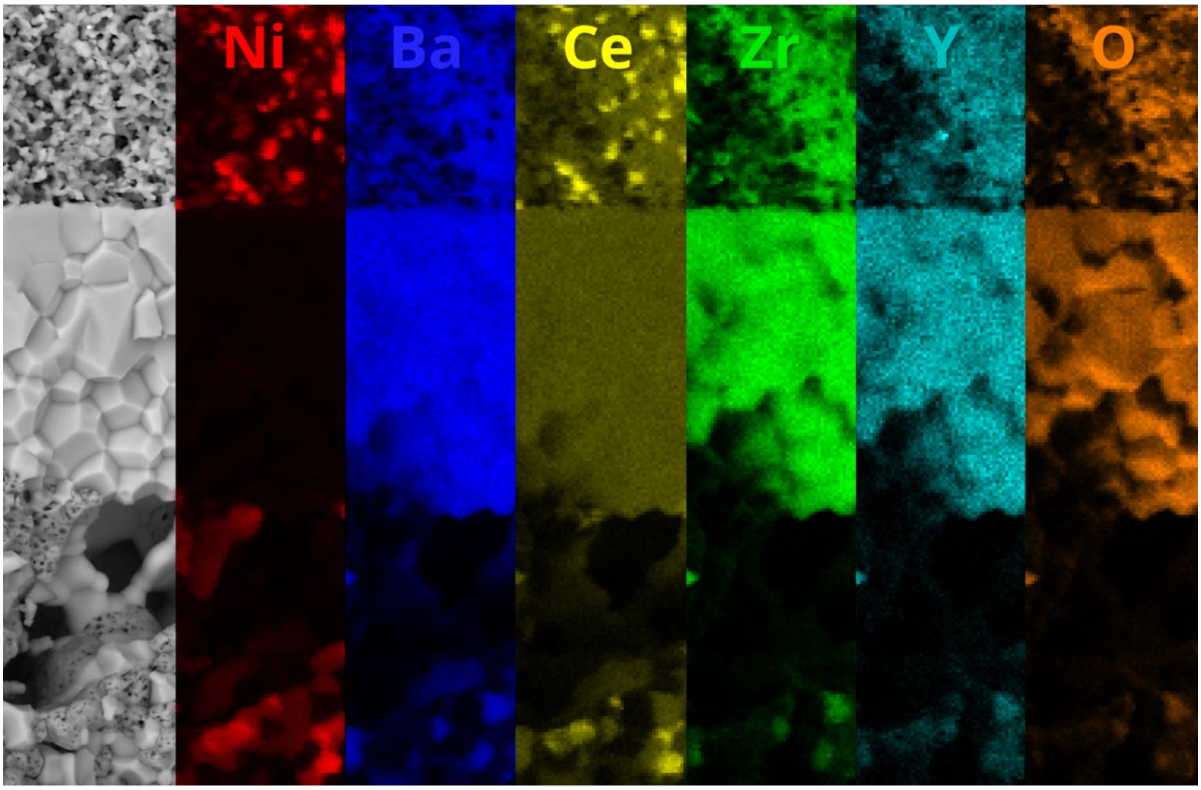

Figure S10: Elemental map analysis on the fractured cross-section surface of a fully reduced P-SOC. Notably, the absence of nickel within the electrolyte depicts the integrity during high sintering temperature. The occurrence of ceria highlights are coinciding with that of the nickel in both the electrodes. Since the emission energies of ceria M_a (0.88 keV) and M_b (0.90 keV) occur at the tail of nickel peak at L_{a1} (0.85 keV) and L_{b1} (0.87 keV) during the mapping, the software registers ceria at nickel locations as well.

Kramers-Kronig residuals and equivalent circuits.

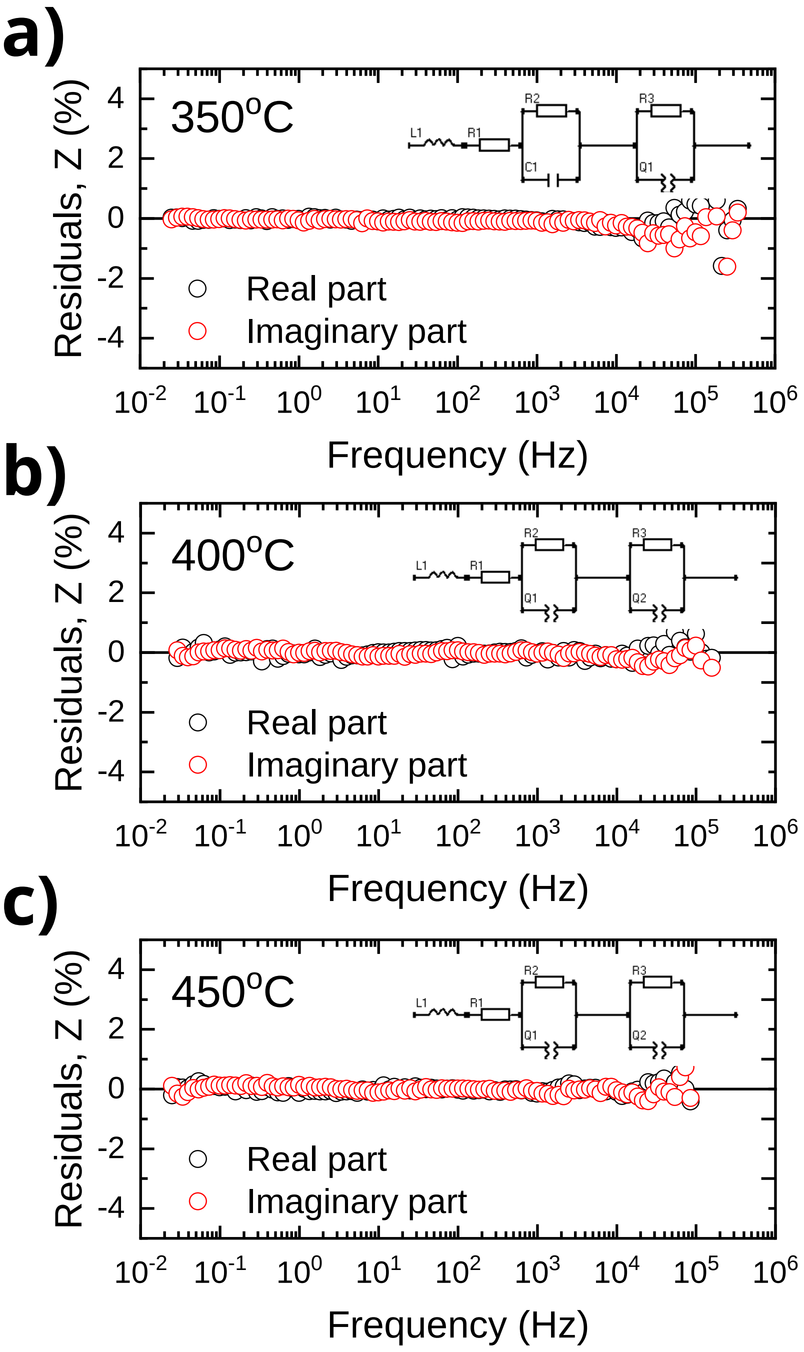

Figure S11: KK-test residuals for the EIS measurements performed at a) 350, b) 400 and c) 450 °C under humidified hydrogen (10 vol.% H_2) in helium at negatrode | humidified hydrogen (60 vol.% H_2) in helium at positrode. Inserts for a depiction of suitable equivalent circuits.

Aging IV and EIS plots

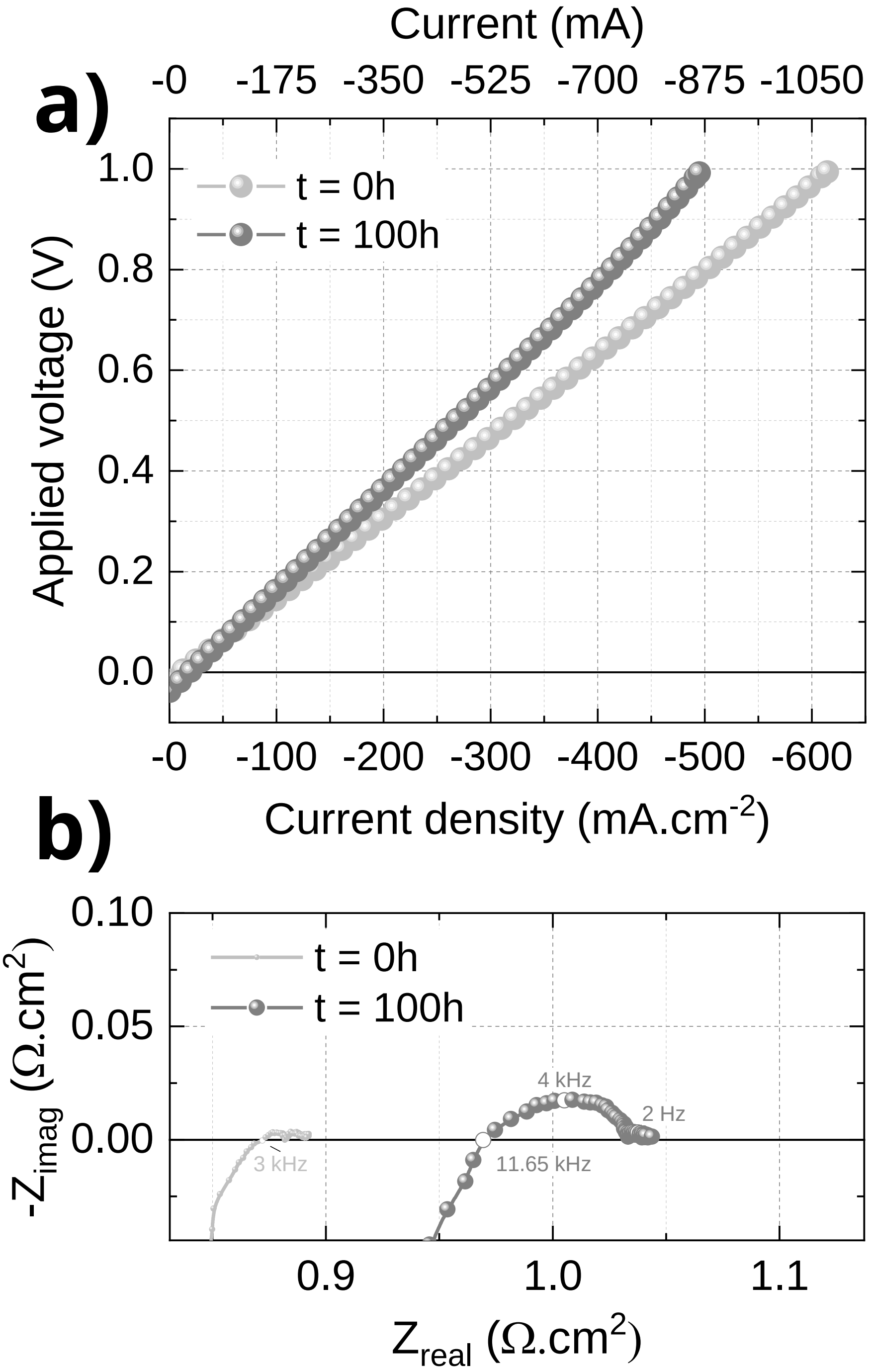

Figure S12: State of the P-SOC before and after aging for 100 h at 320 mA.cm^{-2} at 450 °C under humidified helium at negatrode | humidified hydrogen (60 vol.% H_2) in helium at positrode. a) I-V polarization characteristics as hydrogen pump, b) electrochemical impedance spectra.

Post aging test SEM

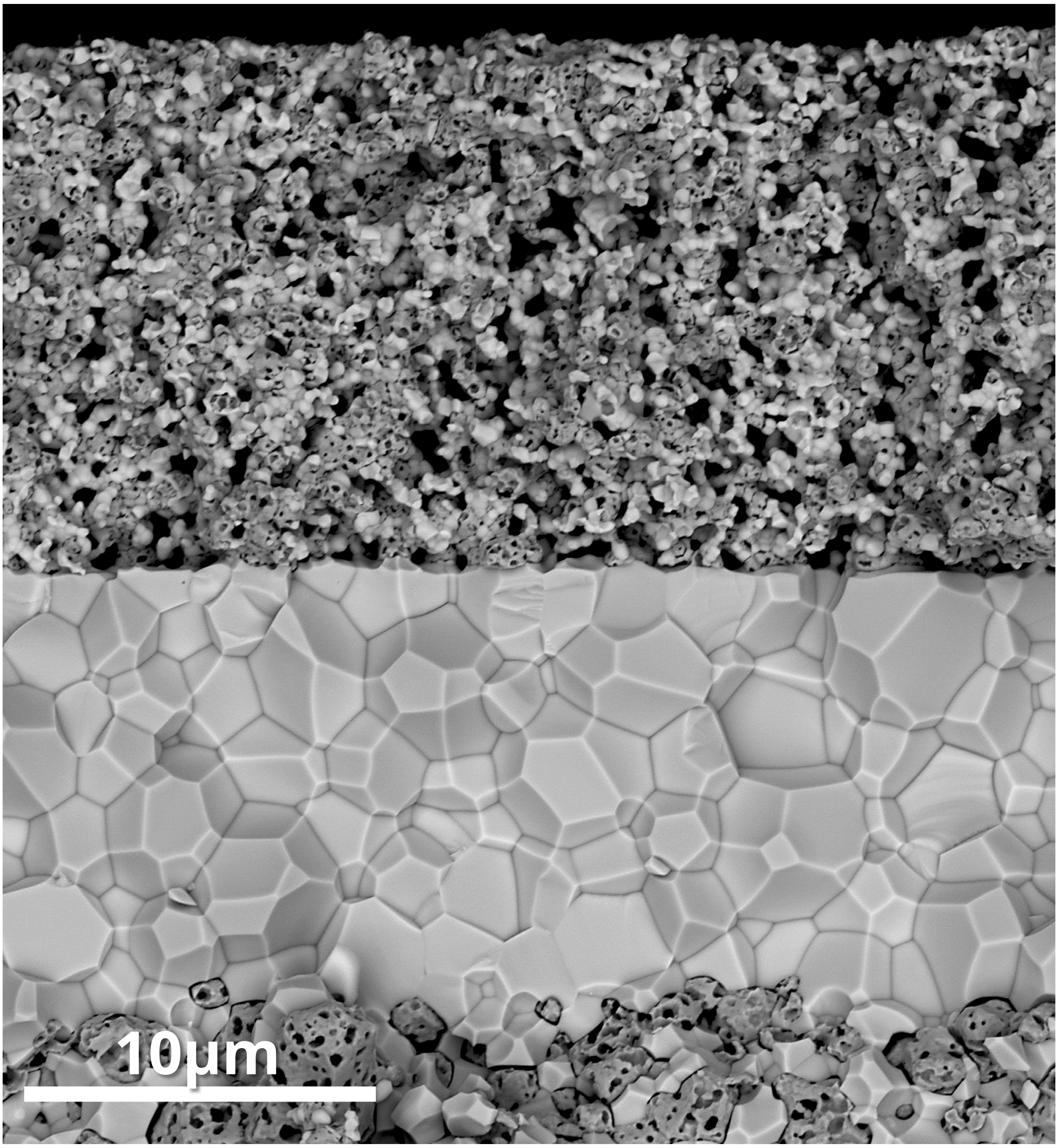

Figure S13: SEM cross-section image of the P-SOC after ageing for 100 h at 320 mA.cm^{-2} at 450 ºC under humidified helium at negatrode | humidified hydrogen (60 vol.% H_2) in helium at positrode. There are no signs of electrode delamination or nickel migration away from the interface except further generation of microporosity which could be attributed to the cause of initial degradation.
